# Evidence, detailed characterization and clinical context of complement activation in acute multisystem inflammatory syndrome in children

**DOI:** 10.1038/s41598-022-23806-5

**Published:** 2022-11-17

**Authors:** György Sinkovits, János Schnur, Lisa Hurler, Petra Kiszel, Zita Z. Prohászka, Pál Sík, Erika Kajdácsi, László Cervenak, Veronika Maráczi, Máté Dávid, Borbála Zsigmond, Éva Rimanóczy, Csaba Bereczki, Loek Willems, Erik J. M. Toonen, Zoltán Prohászka

**Affiliations:** 1 Department of Internal Medicine and Hematology, Semmelweis University, Budapest, 1085 Hungary; 2 Heim Pál National Pediatric Institute, Budapest, 1089 Hungary; 3 Research Group for Immunology and Hematology, Semmelweis University-Eötvös Loránd Research Network (Office for Supported Research Groups), Budapest, 1085 Hungary; 4 Department of Pediatrics, University of Szeged, Szeged, 6720 Hungary; 5 R&D Department, Hycult Biotech, 5405 PB Uden, The Netherlands

**Keywords:** Complement cascade, Biomarkers, Immunological disorders

## Abstract

Multisystem inflammatory syndrome in children (MIS-C) is a rare, life-threatening complication of severe acute respiratory syndrome coronavirus 2 (SARS-CoV-2) infection. MIS-C develops with high fever, marked inflammation and shock-like picture several weeks after exposure to, or mild infection with SARS-CoV-2. Deep immune profiling identified activated macrophages, neutrophils, B-plasmablasts and CD8 + T cells as key determinants of pathogenesis together with multiple inflammatory markers. The disease rapidly responds to intravenous immunoglobulin (IVIG) treatment with clear changes of immune features. Here we present the results of a comprehensive analysis of the complement system in the context of MIS-C activity and describe characteristic changes during IVIG treatment. We show that activation markers of the classical, alternative and terminal pathways are highly elevated, that the activation is largely independent of anti-SARS-CoV-2 humoral immune response, but is strongly associated with markers of macrophage activation. Decrease of complement activation is closely associated with rapid improvement of MIS-C after IVIG treatment.

## Introduction

Multisystem inflammatory syndrome in children (MIS-C) is a rare (5.1/1 million person-months in the general population; and 316/1 million person-months in SARS-CoV-2 infected), severe, potentially life-threatening complication of SARS-CoV-2 infection^1^. MIS-C typically occurs in 8- to 12-year-old children several weeks after exposure to, or infection with SARS-CoV-2, even if the disease was asymptomatic^2^. Presenting clinical features include fever, rash, mucositis, conjunctivitis, cardiac complications, and hypotension or shock. Almost all of the affected cases are anti-spike IgG seropositive indicating reconvalescent stage, but most of them are viral RNA negative in nasal swabs making viral persistence less plausible as a trigger of the condition. The optimal treatment of MIS-C is currently unknown, intravenous immunoglobulin (IVIG) preparations (with or without corticosteroids) are widely used to good effect, rapidly leading to resolving symptoms.

Initial multi-omic studies described deep immune profiles and the disease landscape in MIS-C, and compared them to those of COVID-19 in children. Single-cell RNA sequencing and mass cytometry identified in MIS-C highly activated neutrophils, classical and non-classical monocytes and memory CD8 + T cells with increased frequencies of B-cell plasmablasts^3,4^. Autoimmune profiling showed autoantibody responses to both ubiquitously expressed and tissue specific antigens^5^, against endothelial, mucosal, and immune antigens^4,6^. Serum proteome analysis reported multiple markers of inflammation, humoral immune response, complement and coagulation pathways, together with S100A family alarmins to be enhanced in the samples of MIS-C cases^4,5^. Cytokine release syndrome is dominated by interferon (IFN) gamma^7^ and resembles that of macrophage activation syndrome and endothelial dysfunction^8^.

As early as in the summer of 2020, right after the first wave of COVID-19, complement was implicated to play a role not only in the pathogenesis of severe COVID-19^9^, but also in the pathogenesis of MIS-C. In their initial work, Diorio and colleagues measured the terminal pathway activation marker sC5b-9 in 6 children with MIS-C for the first time and observed a trend for higher values compared to children with mild COVID-19^10^. In the next analysis, the same group reported elevated levels of sC5b-9 in 18 MIS-C cases, comparable to concentrations observed in severe pediatric COVID-19^11^. When analyzed together, no association between anti-SARS-CoV-2 receptor-binding domain (RBD) antibody levels and sC5b-9 was observed, suggesting that anti-SARS-CoV-2 immune complexes are likely not the activating factors behind complement activation in these patients. However, activation of the classical complement pathway was not formally investigated in this study. In their serum proteomic profiling study Porritt et al. observed upregulated heavy and light chains of immunoglobulins and C1QA, C1QB and C1QC proteins in severe MIS-C cases, without further investigation of complement activation in that cohort^5^. The study of Syrimi and co-workers investigated the sC5b-9 level together with other complement protein and regulator levels in 16 cases with MIS-C^3^. Authors observed that C9 and Factor I levels were increased, and the terminal complement pathway was activated in MIS-C, but analysis of potential triggers behind this activation (like anti-SARS-CoV-2-antibodies) was not performed. The role of circulating immune complexes behind complement activation was formally investigated by the group of Hoste et al., who analyzed diluted plasma samples of 10 MIS-C cases and 6 healthy controls. Although a clear trend for increased levels of complement proteins and activation markers was present, statistically significant differences were not observed (except for the elevation of C1-inhibitor)^7^. The above pioneer studies providing the first implications about the potential involvement of complement in the pathogenesis of MIS-C used multi-omic technologies on a few samples to describe the immune landscape of this disease. Detailed analysis of the presence and potential triggers of complement activation, involving all of the pathways, has not been performed until now. Lastly, the effect of treatment on the complement system has not been analyzed in the context of anti-SARS-CoV-2 antibody response.

In order to have a comprehensive view of the role of the complement system in the pathogenesis of MIS-C, we enrolled 34 acute, well-characterized MIS-C cases to this study, and took appropriate samples for detailed complement analysis before therapy. We measured multiple complement component, regulator and activation product levels, allowing us to draw conclusions on the presence and potential causes of complement activation and consumption. Twenty-eight of the 34 acute patients in the cohort were sampled also in remission, making the paired analysis of complement profile changes along disease activity possible. Our results indicate the presence of complement activation in MIS-C with rapid normalization of activation product levels after IVIG treatment.

## Results

### Description of the MIS-C cohort

**Figure 1:**
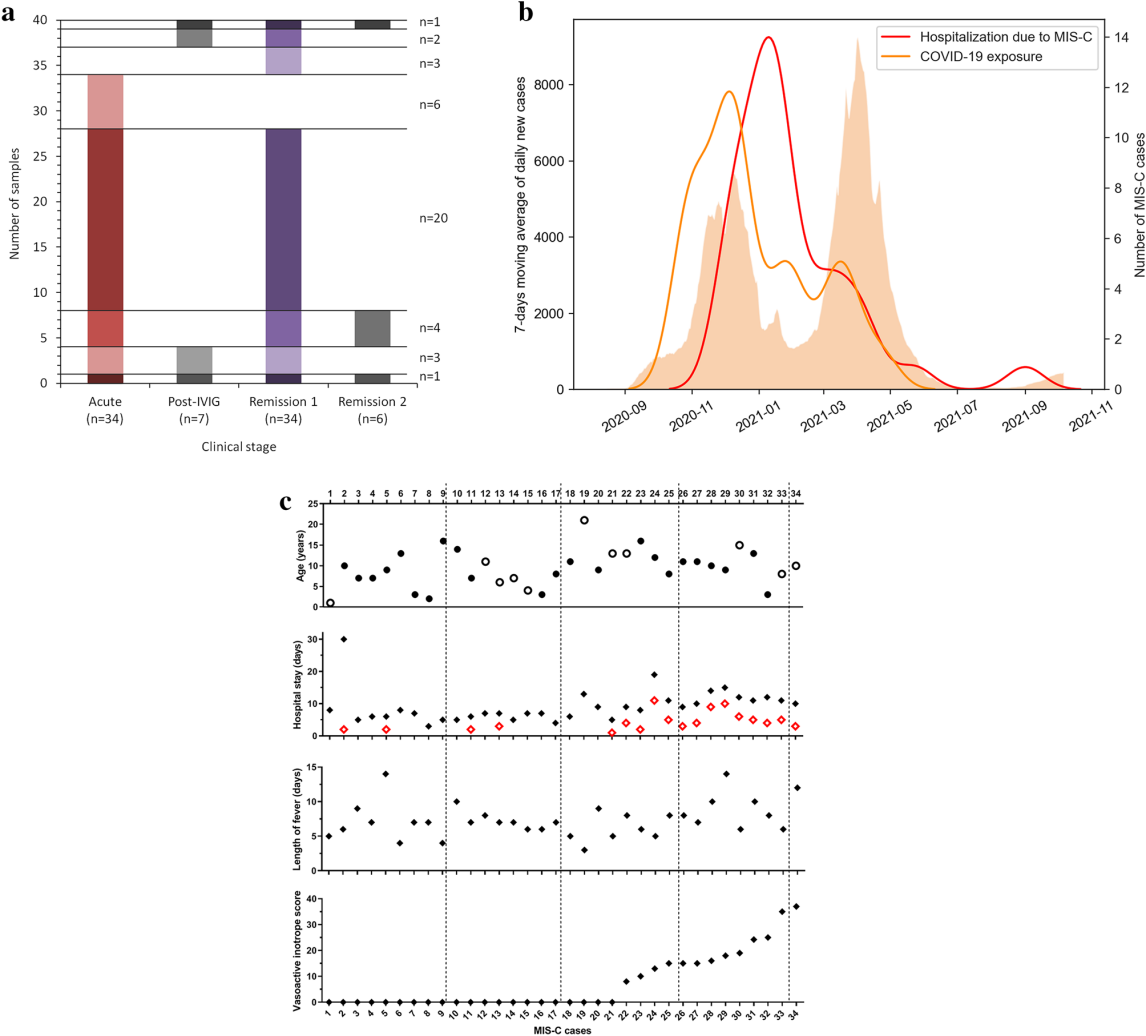
Demographic and clinical data of the MIS-C cohort. (**a**): Study samples included shown by clinical stage. The left axis shows number of cases, case numbers (n) written on the right sum up cases falling into the various sample combination groups. (**b)**: 7-days moving average of daily new COVID-19 cases in Hungary (orange columns, left y axis), as identified by PCR or rapid antigen testing (source: koronavirus.gov.hu, downloaded 27-01-2022). Red line: Kernel density estimation (KDE) plot fitted on the MIS-C cases admitted to the Heim Pál National Pediatric Institute and University of Szeged between 25-11-2020 and 27-08-2021. Orange line: KDE plot fitted on COVID-19 exposures for 24/34 MIS-C cases, for whom information on contact was available. The KDE curves indicate the estimated number of COVID-19 exposures or MIS-C cases, respectively, expressed as cases per 30 days (right axis). (**c**) Age (solid symbols: males, open symbols: females) and disease severity indicators of MIS-C cases (for hospital stay solid diamonds: days hospitalized, open diamonds days in PICU). For calculation of vasoactive inotrope score see the "materials and methods" section.

Between November 2020 and September 2021, we recruited 33 hospitalized children with acute MIS-C at Heim Pál National Pediatric Institute and one patient at the University of Szeged (Fig. 1A). Six additional patients were also enrolled without available sample from the acute stage (post-IVIG and remission samples). All of the patients fulfilled the diagnostic criteria of MIS-C as defined by the World Health Organization (https://www.who.int/publications-detail/multisystem-inflammatory-syndrome-in-children-and-adolescents-with-covid-19). For 24/34 cases exposure (close contact) to COVID-19 patients was documented, MIS-C developed approximately 1 month later (median 31, 25th-75th percentile 24–40, range 3–83 days; Fig. 1B and Supplementary Fig. S1). Ten of the above cases had evidence of a previous symptomatic COVID-19 disease, which started median 27 days (interquartile range: 23–33 days, range: 3–41 days) before the onset of MIS-C. The presence or absence of COVID-19 symptoms was not associated with the severity of MIS-C (see Supplementary Fig. S2). Age distribution of the cohort was between 1 and 21 years, with peak around 10 years (median 9.5, 25th-75th percentile 7–13 years) with male predominance (23/34, 68%, Fig. 1C). Fifty-three percent (18/34, severe group) of the cases required pediatric intensive care unit (PICU) treatment (13/18 PICU cases also required vasopressor and/or inotrope treatment), whereas 47% (16/34) were mild-moderate cases (Fig. 1C). The pediatric healthy control group consisted of 18 children who underwent minor elective surgery (61% males, 8 (median), 6.5–13 (25th-75th percentile), 1–15 (range) years old).

All of the patients received 2 g/kg of body weight intravenous immunoglobulin, 97% of them low-dose aspirin (3–5 mg/kg, max 100 mg), 76% intravenous corticosteroids (2 mg/kg methylprednisolone; 29% of the patients also pulse corticosteroid 2 × 15 mg/kg for three days), and 59% prophylactic low-molecular weight heparin treatment. None of the patients received second line immunomodulatory treatment. All 34 patients survived, 32 patients without long-term sequelae at the time of last follow-up, although one patient with stroke and one with mesenterial thrombosis needed longer follow-up and rehabilitation.

### Macrophage activation and neutrophilia are associated with disease severity in MIS-C

**Figure 2:**
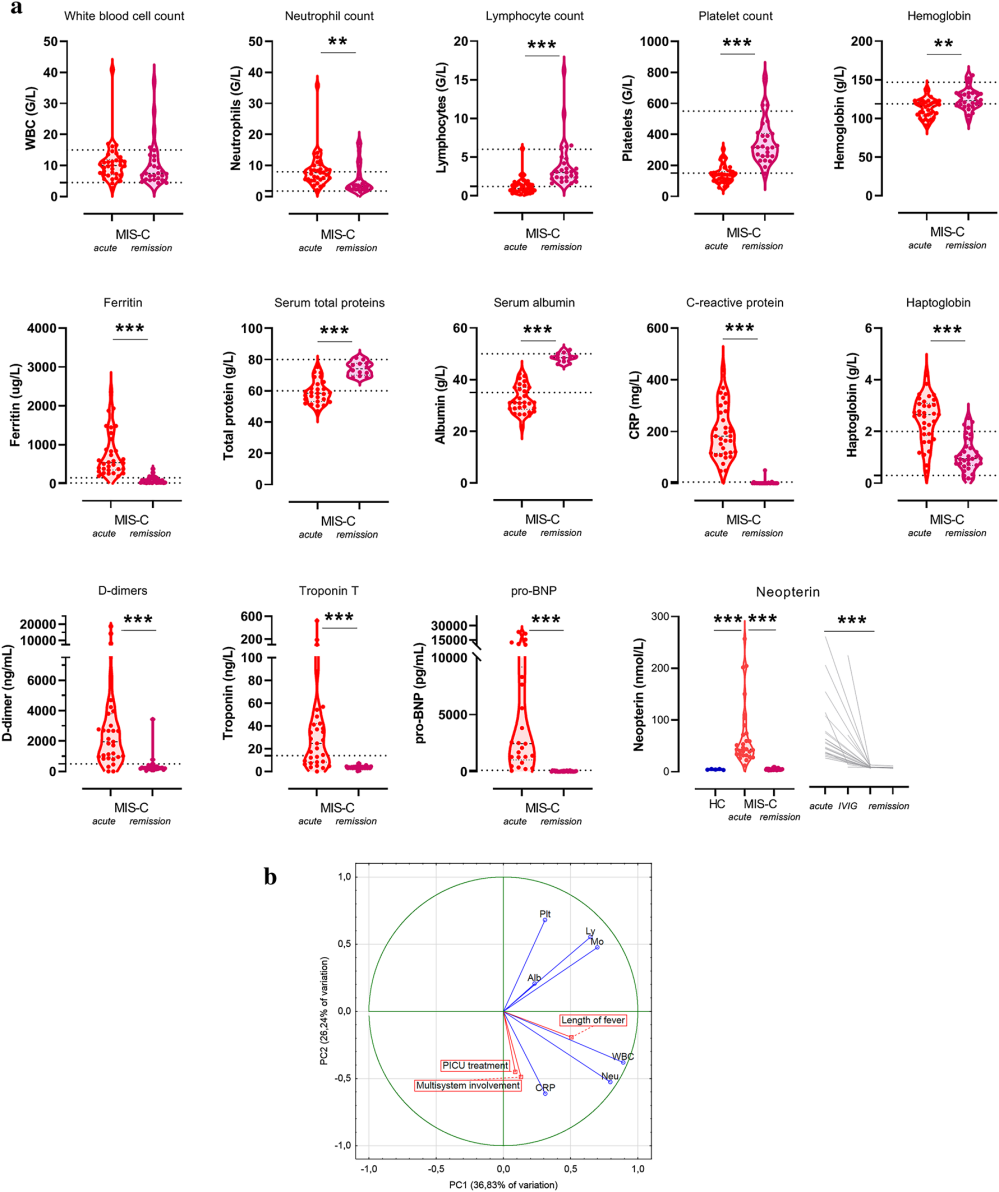

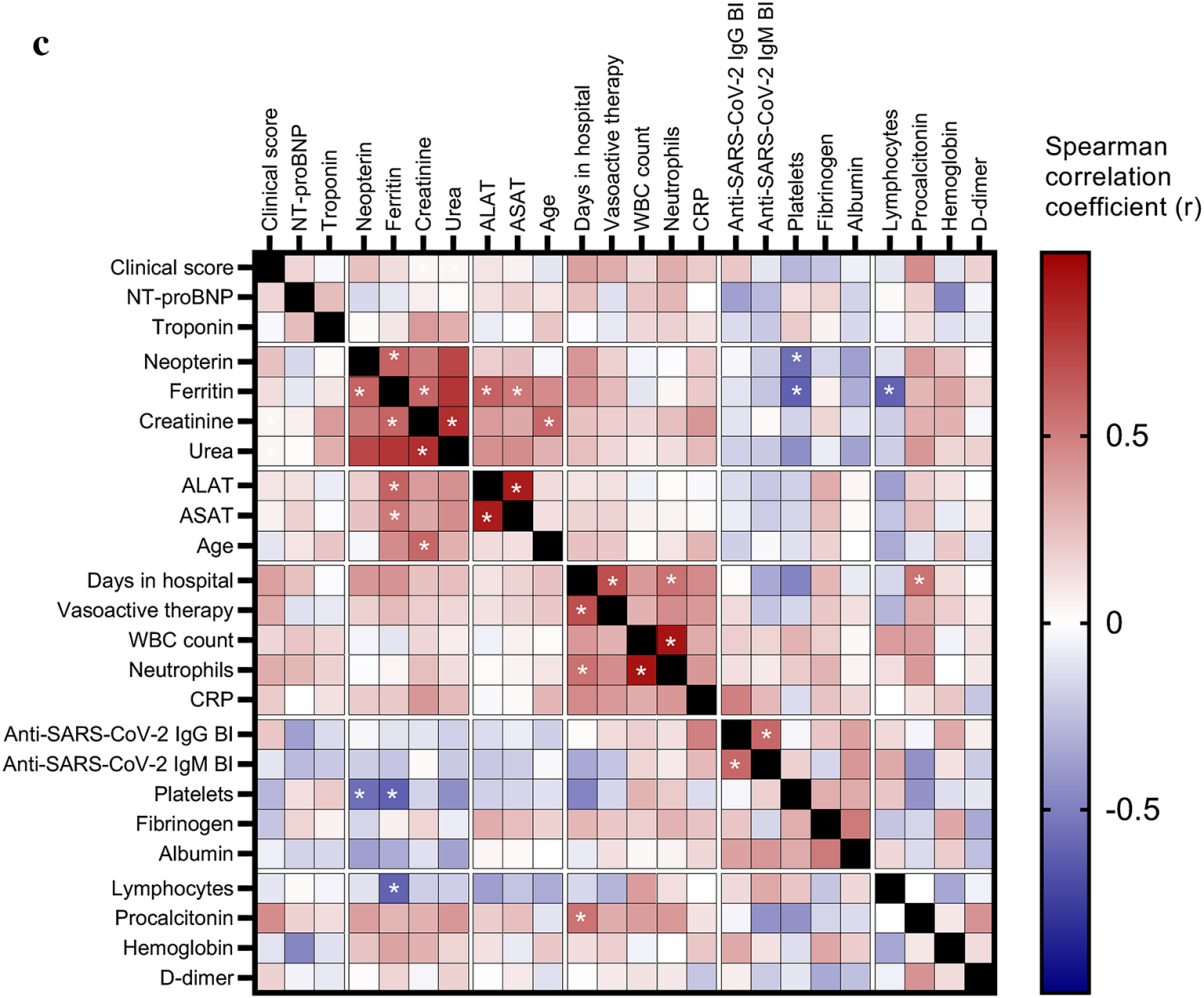
Disease severity marker levels before and after IVIG therapy in the MIS-C cohort. (**a**): Pre- and > 10 days post-IVIG treatment (denoted *acute* and *remission*) absolute blood counts and clinical laboratory results of the 34 MIS-C cases. The horizontal lines indicate the age-specific reference ranges. Since no established reference range was available for neopterin, results of healthy controls are shown for this marker, together with individual changes of levels during IVIG treatment and in remission. P value symbols (**p < 0.01, ***p < 0.001) indicate significant results after 5% false discovery rate correction using the Benjamini–Hochberg method. (**b**): Principal component analysis loading plot of the top 7 features contributing to principal components one and two. Loading of length of fever, PICU treatment and extent of multisystem involvement (indicated by boxes) are shown as supplementary variables. (**c**)**:** Heatmap of correlation matrix of clinical and laboratory markers of MIS-C. Clinical score indicates the total number of organ systems involved, including skin (rash, feet and hands signs), mucosal (conjunctivitis, cheilits, oral mucositis), gastrointestinal (diarrhea, vomiting, abdominal pain) and cardiac involvement. Color-coding indicates the strength of each correlation (Spearman correlation coefficients) with asterisks indicating significance (* indicate *p* < 0.003 significant results, the limit was obtained after 5% false discovery rate correction using the Benjamini–Hochberg method). Non-significant differences are not marked.

Absolute blood cell counts, disease activity, inflammatory and additional specific marker levels are presented on Fig. 2A. When compared to known reference ranges, the majority of the patients had elevated neutrophil and decreased lymphocyte and platelet counts in acute stage. All acute cases showed marked elevations of C-reactive protein and ferritin levels, whereas for most of them haptoglobin, D-dimer, troponin and pro-BNP levels were also elevated, with lower total protein and albumin levels. This clearly supports the presence of severe, systemic inflammation. The most striking difference between healthy children and acute MIS-C cases was noted for neopterin, a marker of macrophage activation^12^. When investigated in remission, after IVIG therapy, all cell counts, inflammatory, cardiac and macrophage activation markers returned to the reference ranges, supporting the presence of full disease remission (Fig. 2A). The normalization of the above markers occurred irrespective of glucocorticosteroid therapy in addition to IVIG (see Supplementary Fig. S3, S4).

With the help of principal component analysis, the overall architecture of severity markers was obtained (Fig. 2B). The first two components, explaining 63.07% of the variance in the data, showed that lymphocyte, platelet, monocyte and albumin levels correlated with each other with CRP level, WBC and neutrophil counts orthogonal to these variables. Interestingly, length of fever, requirement of PICU treatment, and extent of multisystemic involvement clustered together with CRP, WBC and neutrophil counts. Figure 2C illustrates the relationships between clinical severity, cell counts and clinical laboratory markers. Neutrophilia shows association with number of days in hospital, whereas markers of macrophage activation (ferritin^13^ and neopterin^12^) correlate with low platelet and lymphocyte counts and signs of kidney and hepatic involvement. Interestingly, whereas all patients were positive for IgG anti-SARS-CoV-2 antibodies, their levels were not associated with clinical or laboratory markers of MIS-C severity.

### High levels of complement activation markers are characteristic for acute MIS-C and show rapid decline after IVIG therapy

The complement profile was analyzed in a comprehensive manner in this study including measurement of all pathways' activities, components, regulators and specific activation products. This strategy allowed us to conclude on the presence, extent and potential causes of complement activation in MIS-C.

**Figure 3:**
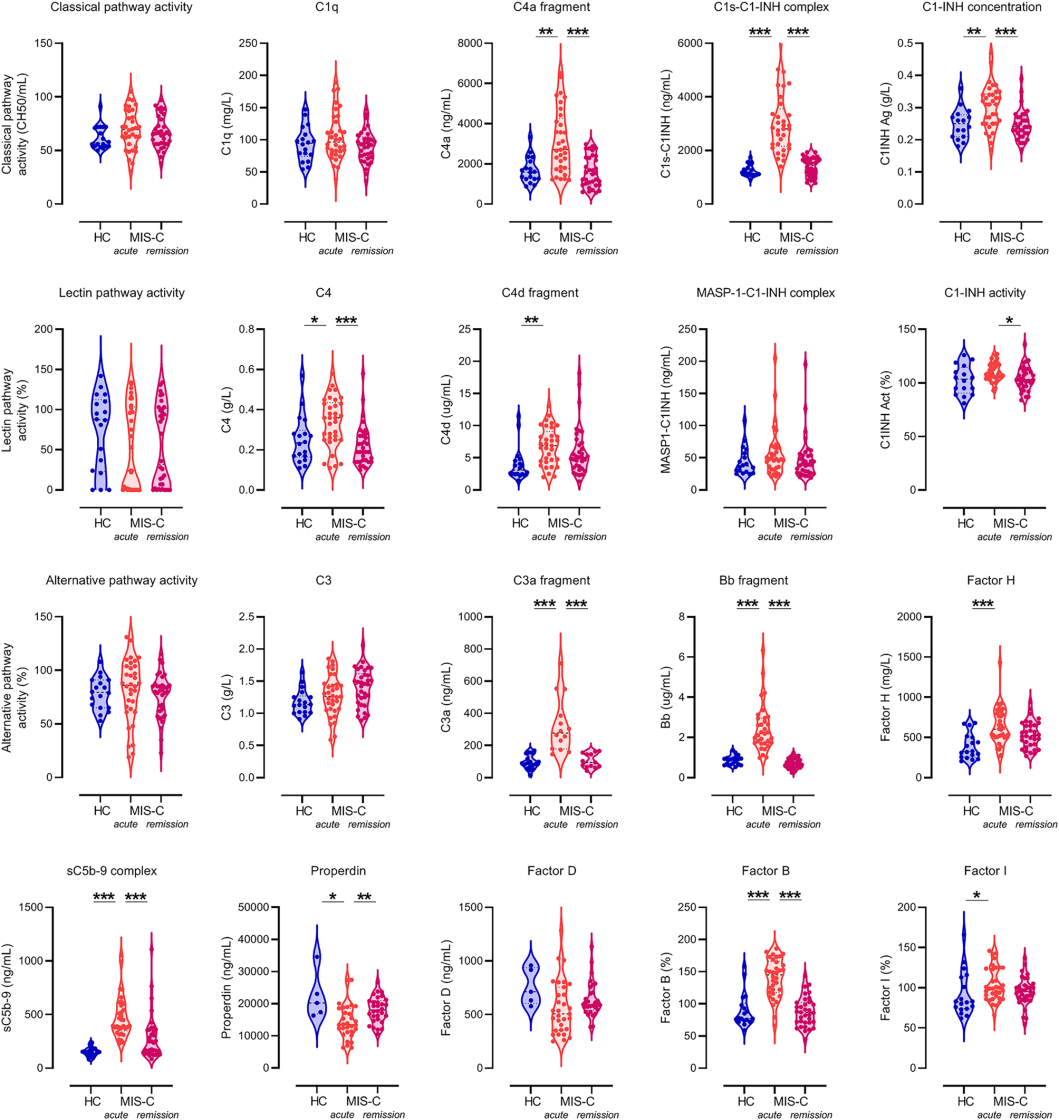
Detailed complement profile of the MIS-C cohort before, during and after IVIG therapy, and of healthy controls. Levels of the complement pathway (classical-, lectin- and alternative) activities, factors (C1q, C4, C3, Factors D, B and properdin) and regulators (C1-inhibitor antigen and activity, Factors H and I) were determined in serum, whereas that of activation products (C1s-C1-inhibitor complex, MASP1-C1-inhibitor complex, C4a, C4d, C3a, Bb and sC5b-9) in EDTA plasma. Violin plots (with dashed horizontal lines indicating the median values, dotted lines indicating the quartiles) show results for the 18 healthy control children (HC), and for all MIS-C cases who had samples from acute stage, before IVIG treatment, or from stable remission after hospital discharge. P values for the pair-wise group comparisons (HC-acute and acute-remission groups) on the violin plots were calculated by the Mann–Whitney test. Non-significant differences are not marked, and p value symbols (*p < 0.05, **p < 0.01, ***p < 0.001) indicate significant results after 5% false discovery rate correction using the Benjamini–Hochberg method.

First, complement markers were compared between 18 healthy control children and 34 MIS-C cases in acute stage, before treatment. As presented on Fig. 3, acute MIS-C patients showed significant elevation of complement components C4 and Factor B, of complement regulators C1-inhibitor, Factor H and Factor I (*p* = 0.0205, *p* < 0.0001, *p* = 0.0026, *p* < 0.0001, and *p* = 0.0225, respectively). Activation products C1s-C1-inhibitor complex, C4a, C4d, C3a, Bb and sC5b-9 were significantly increased (*p* < 0.0001, *p* = 0.0019, *p* = 0.0016, *p* < 0.0001, *p* < 0.0001, and *p* < 0.0001, respectively), whereas lectin pathway activation marker MASP1-C1-inhibitor complex was not. Interestingly, concentration of properdin, the positive regulator of alternative pathway, was decreased, when compared to healthy controls. Functional activities of the lectin, classical and alternative pathways were similar in the two groups, without significant differences in levels of C1q, Factor D and C3. These results support the presence of hypercomplementemia with increased activation product levels of the classical, the alternative and the terminal pathway.

Next, we analyzed changes of the complement biomarker levels in relation to disease stage. All patients received IVIG and went into clinical remission rapidly. Follow-up samples were taken in remission for 34 patients (Fig. 1A), typically 1–2 months after hospital discharge. As shown in Fig. 3, levels of the complement components C4 and Factor B, and the complement regulators C1-inhibitor antigen and properdin concentrations normalized after treatment, as also observed for the activation products C1s-C1-inhibitor complex, C4a, C3a, Bb and sC5b-9. Split product C4d, Factor H and Factor I were the only markers without significant decrease in response to treatment. To give a global view on characteristic changes (direction, extent) of complement biomarkers, line charts in Supplementary Fig. S5 show all measurement points for the individual MIS-C patients (for case/sample numbers see Fig. 1A), with asterisks indicating significance between the 28 acute- first remission sample pairs (paired analysis). This paired analysis shows again that levels of the complement components C4 and Factor B, the complement regulators C1-inhibitor antigen and properdin, and the activation products C1s-C1-inhibitor, C4a, C3a, Bb and sC5b-9 normalized after treatment, with most pronounced and uniform changes for the markers C1s-C1-inhibitor, Bb and C3a. Although activity of C1-inhibitor was not significantly elevated in acute MIS-C samples (when compared to healthy controls, despite the fact that antigenic expression was increased), C1-inhibitor functional activity significantly declined in the MIS-C cohort in response to treatment *p* = 0.024). The comprehensive analysis of the complement biomarkers convincingly shows that remission of MIS-C is characterized by the normalization of the hyperactive complement system, parallel with the decline in additional inflammatory markers (Fig. 2A). The complement profile normalized both in patients who did and in those who did not receive glucocorticosteroids in addition to IVIG (see Supplementary Fig. S6, S7).

For 7 patients, samples taken 2–10 days after IVIG treatment were also available (group 'IVIG' on the line charts of Supplementary Fig. S5). Remarkably, the most rapid, pronounced and uniform decline right after IVIG therapy, was noted for the markers C1s-C1-inhibitor, Bb, C3a and neopterin (Supplementary Fig. S5 and Fig. 2A, line charts). These results indicate that the normalization of complement and macrophage activation marker levels occur rapidly, within days after IVIG treatment.

### Analysis of associations between complement profile, clinical severity, anti-SARS-CoV-2 antibodies, and inflammatory markers

**Figure 4:**
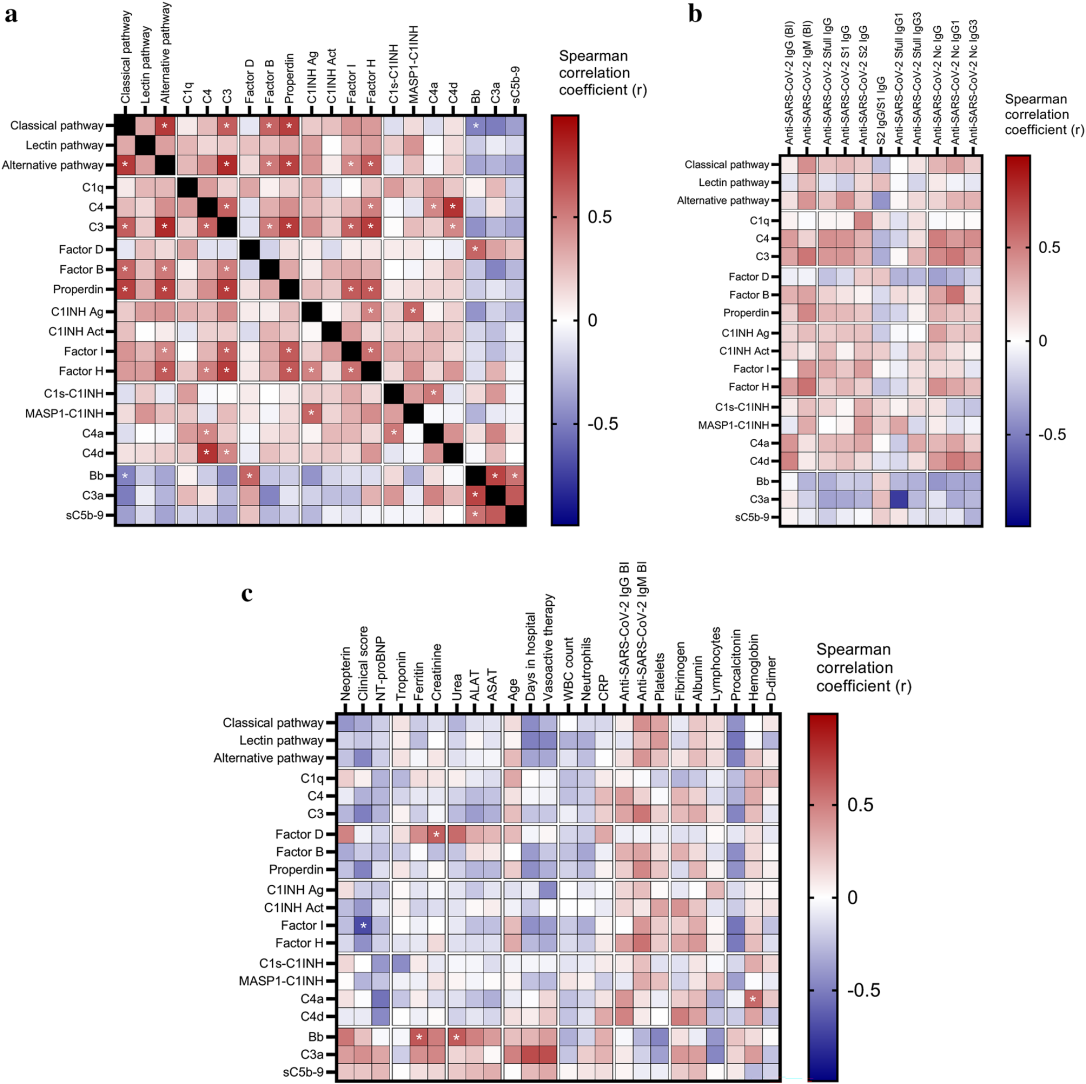
Associations between complement markers, clinical, laboratory and anti-SARS-CoV-2 humoral immune response features in the MIS-C cohort. Heatmap of correlation matrix of complement markers (Panel (**a**), complement markers and clinical and laboratory features (Panel **b**), complement markers and SARS-CoV-2 humoral immune response measures (Panel **c**). Color-coding indicates the strength of each correlation (Spearman correlation coefficients) with asterisks indicating significance (* indicate on Panel (**a**) *p* < 0.007, on Panel (**c**) *p* < 0.005 significant results, the limit was obtained after 5% false discovery rate correction using the Benjamini–Hochberg method).

In addition, the design of our study allowed investigation and identification of potential triggers and interactions underlying complement overactivation in acute MIS-C. We observed that levels of C4, C3, properdin, Factor I, Factor B and Factor H show positive correlations with functional activities of the classical and alternative pathways (Fig. 4A). Similarly strong positive associations were observed between activation markers of the alternative pathway (Bb), all pathways (C3a) and the terminal pathway (sC5b-9), indicating activation of the alternative pathway as the most closely related factor behind terminal pathway activation. Intriguingly, the early classical pathway marker C1s-C1-inhibitor complex level did not show any strong association with the functional activity of the classical pathway, or with the concentration of its specific component C1q. Split product C4a is the only factor whose level correlates with that of the C1s-C1-inhibitor complex (Fig. 4A).

**Figure 5:**
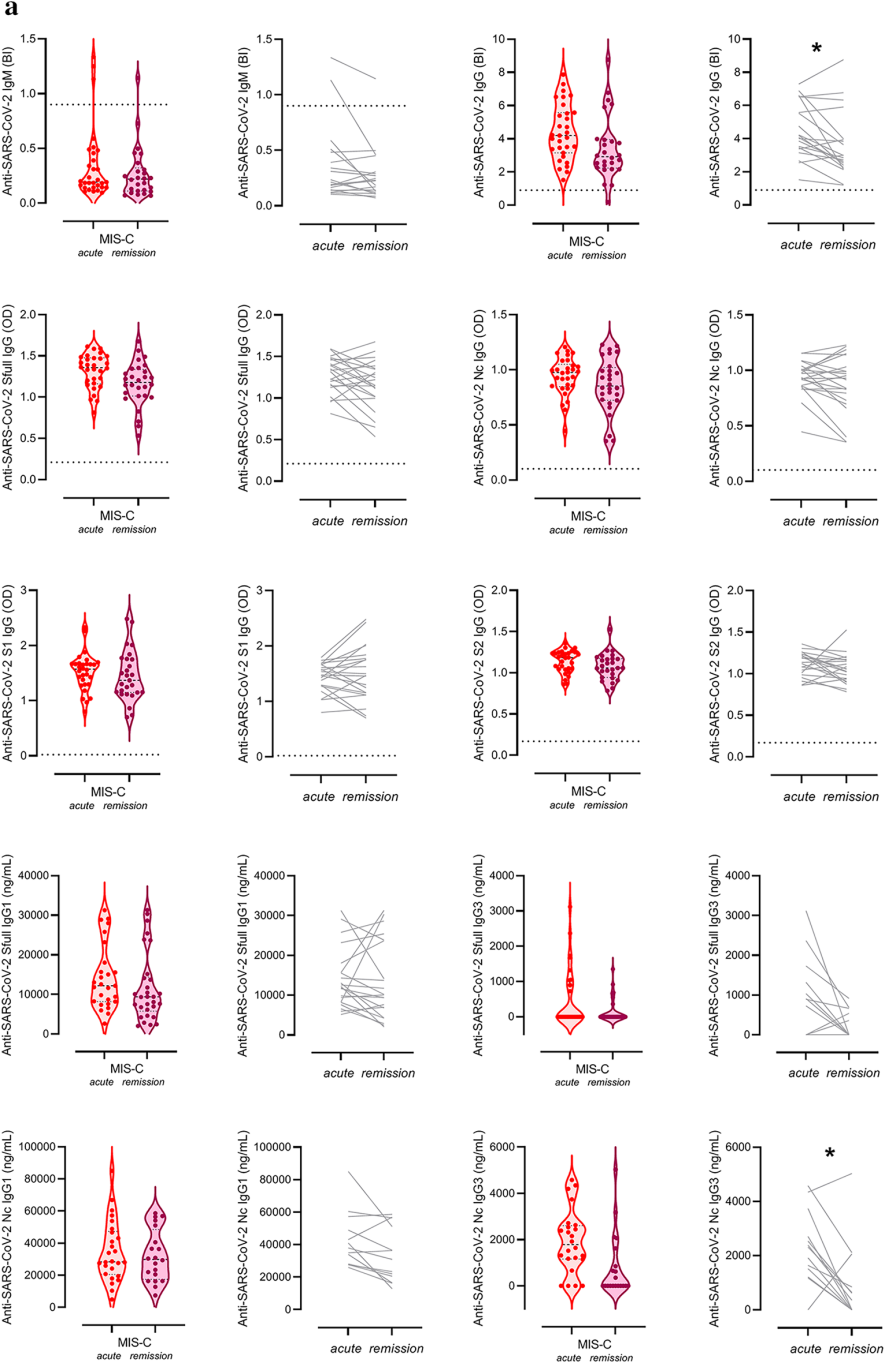

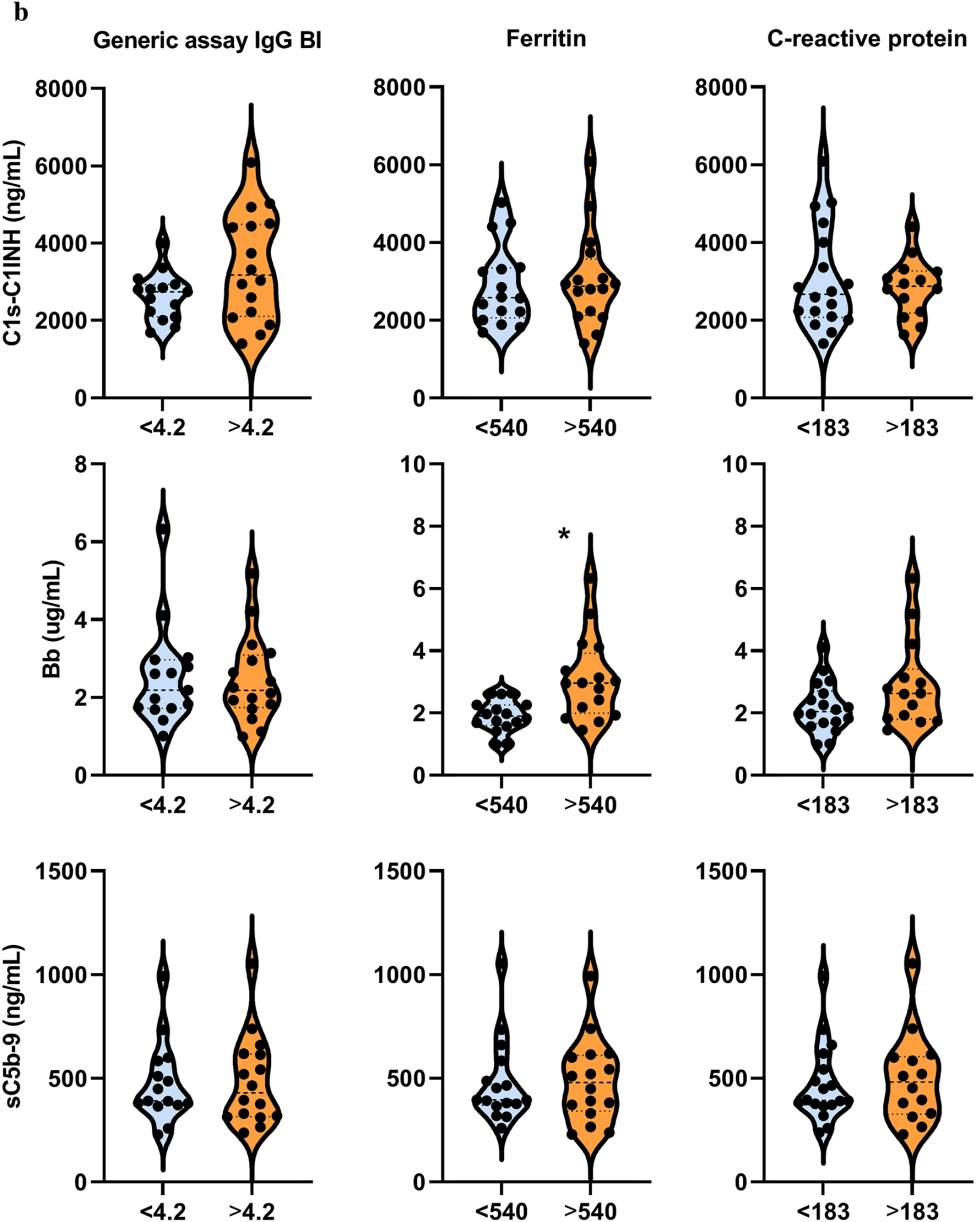
Association between biochemical markers of complement and anti-SARS-CoV2 antibody response, macrophage activation, acute phase reaction and clinical severity of MIS-C. (**a**)**:** Violin plots (with horizontal lines indicating the median values) show results for the MIS-C cases that had samples from acute stage, before IVIG treatment, or from stable remission after hospital discharge. Grey lines show changes of marker levels for individual MIS-C cases from before treatment (*'acute*') after hospital discharge in remission ('*remission*"). P values for the line charts were obtained by the Wilcoxon matched-pairs ranked sum test. Non-significant differences are not marked, and p value symbols (*p < 0.05, **p < 0.01, ***p < 0.001) indicate significant results after 5% false discovery rate correction using the Benjamini–Hochberg method. (**b**): Violin plots showing classical- (C1s-C1-inhibitor complex), alternative- (Bb fragment) and terminal pathway (sC5b-9 complex) activation marker levels in groups with low (below median) or high (above median) anti-SARS-CoV-2 spike and nucleocapsid IgG levels (Generic assay IgG binding index), macrophage activation (ferritin levels) or acute phase reaction (CRP concentration). * indicates *p* = 0.002, considered significant after 5% false discovery rate correction using the Benjamini–Hochberg method.

Intriguingly, there was no significant correlation between any of the complement components, regulators or activation products and features of the anti-SARS-CoV-2 humoral immune response including total IgG and IgM response (against spike (S) + nucleocapsid (Nc) protein antigens), or specific measures of anti-S, anti-Nc total IgG, or specific subclasses (IgG1 and IgG3) (Fig. 4B). It is noteworthy that anti-SARS-CoV-2 antibodies show no significant decline in response to IVIG treatment, except significant, non-uniform decrease of IgG binding index and anti-N IgG3 levels in remission (*p* = 0.0094 and *p* = 0.0134, respectively) (Fig. 5A). These observations make it unlikely that anti-SARS-CoV-2 antibodies play a significant role as trigger factors behind complement activation in acute MIS-C.

Finally, clinical and laboratory markers of MIS-C severity and activity were investigated for their relationship with complement biomarkers in the acute disease stage. As shown on Fig. 4C, the global overview indicates rather low level, if any, of correlations between these factors. Only the markers Bb, C3a and sC5b-9 show a clear trend towards positive correlation with disease severity, including the significant positive correlation between Bb and ferritin and urea levels (*p* = 0.0003 and *p* < 0.0001, respectively). In order to formally explore the potential contribution of key pathogenic features of MIS-C to the elevation of complement activation product levels, selected biomarkers of humoral anti-SARS-CoV-2 immune response, macrophage activation and hepatic acute phase reaction were further investigated (Fig. 5B). When patient groups are stratified according to the median levels of anti-SARS-CoV-2 IgG, ferritin or CRP levels, it is the alternative pathway activation marker Bb that shows a significant association with high levels of ferritin (*p* = 0.0017). The results collectively indicate that macrophage activation and complement activation and amplification via the alternative pathway (C3a) with subsequent formation of the cell activating or damaging sC5b-9 complex are present in parallel, as part of the complex multisystem inflammatory response in MIS-C.

### Ranking of complement and macrophage measures as markers of acute MIS-C and therapy response

**Figure 6:**
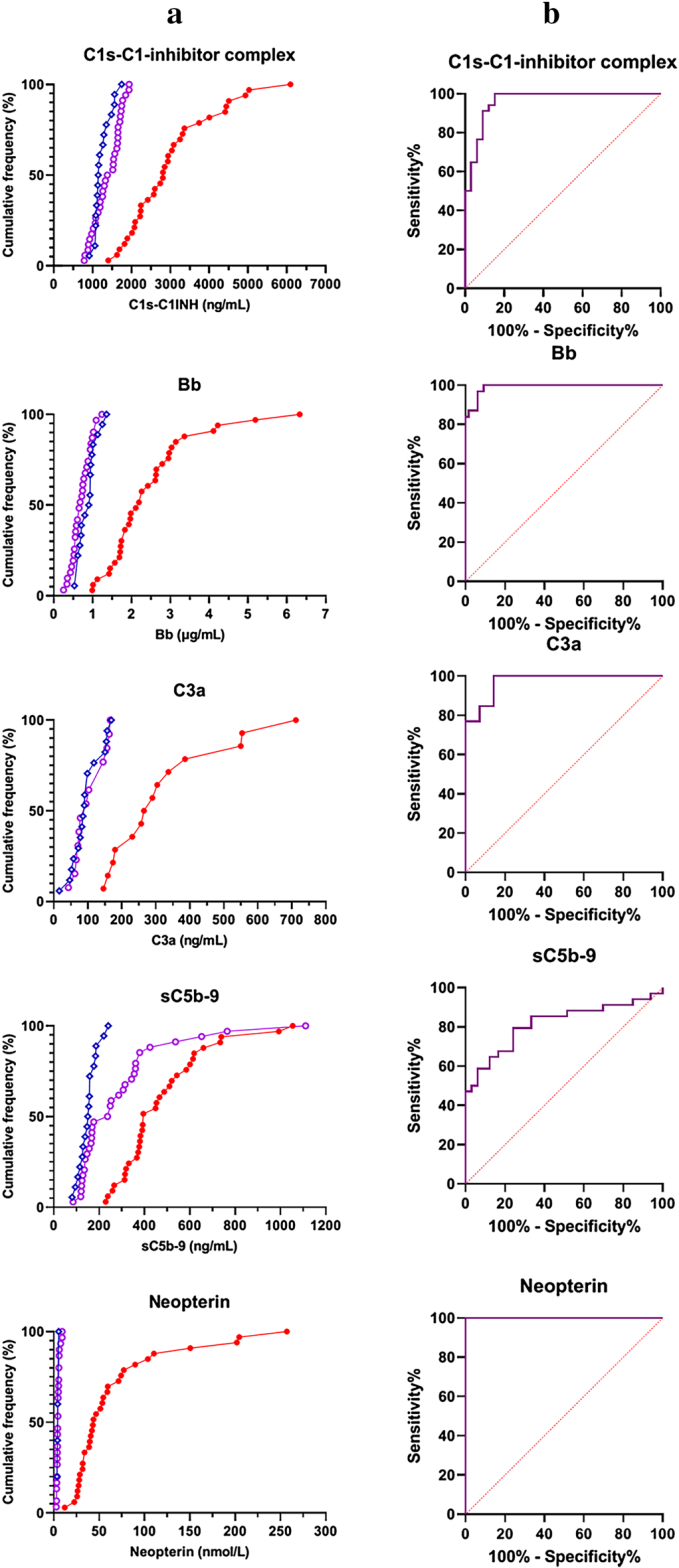

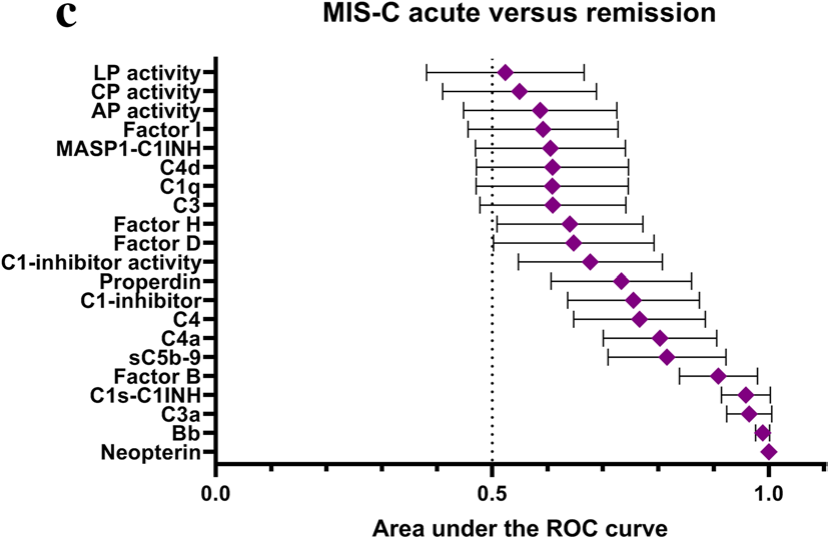
Ranking of complement and macrophage measures as markers of acute MIS-C and therapy response (**a**): Cumulative frequencies (%) of MIS-C cases in acute (red, solid circles) or in remission (purple, open circles) stage, and of healthy controls (blue, open rhombus), plotted against the range of selected complement- and macrophage activation markers. (**b**)**:** Performance of complement activation marker and neopterin levels to describe therapy responses in MIS-C (ROC-analysis). (**c**)**:** Area under the curve (AUC) values with 95% confidence intervals.

Lastly, we performed an analysis of the complement and macrophage measures with the objective to identify the markers that show the closest association with acute MIS-C, or characteristic changes after therapy. For this purpose cumulative frequencies of MIS-C cases and healthy controls were plotted against biomarker levels on Fig. 6A. The same markers were also compared in acute versus remission MIS-C cases. Individual receiver operating characteristic (ROC) curves are presented on Fig. 6B, where the best markers with highest area under the ROC curve (AUC) values were neopterin, C1s-C1-inhibitor and Bb. All these markers had an AUC value near 1 (Fig. 6C, Table 1). These results make the use of the aforementioned macrophage and complement activation markers promising for therapy monitoring purposes.

**Table 1:** Characteristics of selected biomarkers for therapy monitoring in MIS-C.

Marker	Cut-off point	Sensitivity	Specificity	Likelihood ratio*
**Comparison of acute MIS-C cases to remission (monitoring of therapy efficacy)**				
Early classical pathway activation marker C1s-C1-inhibitor complex	1795 ng/mL	91.18 77.04–96.95	90.91 76.43–96.86	10.029 (3.392–29.651)
Alternative pathway activation product Bb	1.11 ug/mL	96.77 83.81–99.83	93.94 80.39–98.92	15.968 (4.161–61.274)
Anaphylatoxin C3a	170 ng/mL	100 77.19–100	85.71 60.06–97.46	7.000 (1.940–25.255)
Terminal pathway activation product sC5b-9	363 ng/mL	79.4 63.20–89.65	75.76 58.98–87.17	3.276 (1.750–6.132)
Macrophage activation marker neopterin	10.7 nmol/L	100 88.65–100	100 89.57–100	inf (nan-inf)

*Likelihood ratio was calculated as sensitivity/(1-specificity), i.e. true positivity rate divided by the false positivity rate, indicating the diagnostic value of having a positive test result in supporting a certain condition (the remission of MIS-C).

For calculation of likelihood ratio positive result was considered in the following way: laboratory parameters below the cut-off were regarded as positive, as these values are supposed to indicate remission, which is the condition in question. Confidence intervals were calculated according to the method described by Simel et al^44^.

*nan* not a number (cannot be calculated); *inf *infinity.

## Discussion

MIS-C is a rare, clinically and temporally distinct manifestation of COVID-19 in children and adolescents^1,2^. Current case definition of this condition relies on the co-occurrence of fever, clinical signs including skin-, cardiac- or gastrointestinal involvement, coagulopathy, hypotension or shock, and evidence of inflammation in SARS-CoV-2 exposed or infected patients under age of 21 (https://www.who.int/news-room/commentaries/detail/multisystem-inflammatory-syndrome-in-children-and-adolescents-with-covid-19). Recent studies show the presence of immune dysregulation in MIS-C including T lymphocyte depletion and activation, T cell receptor Vbeta skewing, elevated plasmablast frequencies, increased markers of vascular pathology, and decreased numbers and functional profiles of antigen-presenting cells^14^. Initial clinical laboratory workup and plasma proteomic analysis identified multiple inflammatory, coagulation, macrophage activation and complement markers to be associated with acute MIS-C^3,8^. Current treatment of MIS-C includes supportive care with, for example, vasoactive medication, in combination with immunomodulatory treatment. The most frequently used agent is IVIG with or without corticosteroids, resulting in rapid resolution of inflammation after therapy^15^.

We performed detailed profiling of the function and activity of the complement system to unravel its role in the pathophysiology of MIS-C. We identified several complement activation products as appropriate markers to verify the clinical diagnosis of MIS-C, and to show therapy efficacy after IVIG treatment. We describe that acute MIS-C is characterized by elevations of C1s-C1-inhibitor complex, C4a and C4d levels, activation markers of the C1 complex and of the common section of the classical and lectin pathways, by increased Bb and C3a concentrations, indicating amplification via the alternative pathway and activation of the central component C3, and finally, by elevation of sC5b-9, the activation product of the potentially cell-damaging terminal pathway. All of these activation product levels (except C4d) showed rapid and sharp decline after treatment by IVIG, together with additional markers of inflammation (Supplementary Fig. S5). These results indicate that complement activation is part of the systemic inflammatory response in MIS-C with rapid resolution after the immunomodulatory therapy. We showed that levels of the inflammatory and macrophage activation marker ferritin was associated with complement activation products Bb, C3a and sC5b-9 suggesting a potential interaction between the two processes in the development of MIS-C.There was no relationship between anti-SARS-CoV-2 humoral immune response features and activation of the classical pathway, that may be explained by the fact that at the time-point of sampling no active SARS-CoV-2 infection was present, hence, viral antigen–antibody immune complexes were also not present. However, these results may also indicate that factors other than SARS-CoV-2 specific immunoglobulins might give rise to the elevation of C1s-C1-inhibitor complex. We also revealed that neopterin, a small molecule marker belonging to the group of pteridines, shows close association with acute MIS-C, and with resolution of inflammation.

Our comprehensive analysis of the complement system in of MIS-C provides new insights into the pathogenesis of this novel disease. Since our study enrolled a high number of acute and remission MIS-C cases, together with healthy controls, we were able to identify the potential causes, mechanism and context of complement activation. First, we showed that multiple complement activation markers are considerably elevated in acute MIS-C, not only terminal pathway marker sC5b-9 as suggested by previous studies^3,8,10,11^. The close association of sC5b-9 with both C3a and Bb makes alternative pathway activation and amplification the likely trigger of terminal pathway activation. Our observations on the activation of the terminal pathway are in complete agreement with the results of Syrimi et al., although sC5b-9 was the only activation marker measured in that study^3^. Second, the prototypic macrophage activation marker ferritin and alternative pathway marker Bb were both elevated and showed a significant association in acute MIS-C (Figs. 4C and 6), with rapid and uniform decrease after immunomodulatory therapy. The results may indicate that alternative pathway and macrophage activation are parallel and/or related processes in MIS-C. Third, no correlation between any of the complement activation markers, including all markers of the classical pathway, and features of the anti-SARS-CoV-2 humoral immune response was observed. Together with the lack of clinically meaningful decline of anti-SARS-CoV-2 antibodies after immunomodulatory therapy (acute and remission stage, grey line charts on Fig. 5A), these observations make it unlikely that anti-SARS-CoV-2 antibodies play a major role in the initiation and sustainment of multisystemic inflammation in these patients. Fourth, it is intriguing that complement factors and regulators C4, Factor B, C1-inhibitor and C1s, which were found to be significantly elevated in acute MIS-C in our current study, were all reported to respond with increased gene expression to interferon gamma and LPS stimuli in mouse macrophage models^16,17^.

A key aim of our study was to explore the potential causes and mechanisms of complement activation in MIS-C. The observation on the lack of association between activation markers of the classical pathway and features of the anti-SARS-CoV-2 humoral immune response is in agreement with the recent observations of Hoste et al.^7^. Authors observed no relationship between non-SARS-CoV-2 specific circulating immunocomplexes (CICs) and complement activation (C4a and sC5b-9), but early activation marker C1s-C1-inhibitor was not investigated. Although in the study of Hoste et al. multiple complement activation markers were determined and partly showed to be elevated, they failed to observe significant differences between acute MIS-C and healthy controls. Potential explanations for this observation might include the facts that 50% diluted plasma samples (collected from whole blood for cell separation purposes) were used, and samples of only 10 MIS-C and 6 healthy control subjects were included in the measurements. In contrast, to the best of our knowledge, our study enrolled the largest number of patients and controls reported until today for complement measurements in MIS-C, and we used appropriately processed and stored EDTA plasma samples for activation product measurements.

We observed that multiple activation markers, not only the terminal pathway marker sC5b-9, are elevated in acute MIS-C and show rapid decline in parallel to additional inflammatory, coagulation and macrophage activation markers. We observed significant associations between the alternative and terminal pathways' activation and macrophage activation markers. Elevation of interferon gamma^7^, and translocation of intestinal lipopolysaccharide (LPS)^18^ have been described as pathogenic features in MIS-C. Both of these factors are well known activators of macrophages with increased expression of components of the classical pathway, alarmins and IFN-induced genes, and were reported to contribute to the development of severe MIS-C^7,8^. Furthermore, terminal pathway activation marker sC5b-9 was also described as an inflammatory trigger for macrophages^19^, and was shown to promote microglia activation by up-regulation of costimulatory molecules and to increase cytokine secretion^20^. Therefore, these results may support the hypothesis that activated macrophages may represent an important source of complement proteins including C1 inhibitor, Factor B and C4, as reported previously in in vitro mouse models^16^. Activation of both C4 and Factor B (elevated C4a, C4d, Bb, but not MASP1-C1-inhibitor complex, Fig. 3) was indeed demonstrated in our study, supporting our conclusion on the activation of both classical and alternative pathways in MIS-C. Further studies are necessary to validate our results, and to explore the detailed mechanism how macrophage activation may lead to the initiation of classical and alternative pathways in this condition.

In search of appropriate and sensitive markers of macrophage activation in MIS-C, we found that ferritin and neopterin levels were highly significantly elevated in the acute disease stage, and showed a uniform sharp decline parallel with the resolution of the hyperinflammatory syndrome. As a surrogate marker for the status of iron storage in the human body, ferritin was originally implicated to play a role in the iron homeostasis of the body^21^. Later on, it was recognized that ferritin is an acute phase protein and a sensitive marker of infections and macrophage activation^22^. Recently, as reviewed by Mahroum et al., ferritin has been recognized as a marker of COVID-19 severity and outcome^23^, and early observational studies on MIS-C described ferritin as a marker of this condition as well^2,24,25^. In contrast, levels of neopterin, a member of the chemical group named as pteridines, which has also been reported to correlate with COVID-19 severity, has never been investigated in MIS-C before^26^. Elevated neopterin levels were observed after non-thermal injury and sepsis, parallel to macrophage activation and decrease of their HLA DR expression^27^, a key feature also reported for MIS-C cases^7,28^. It is noteworthy that neopterin is synthesized by macrophages in response to interferon gamma^12,29^, and indeed showed highest and most uniform difference in acute stage of MIS-C, when compared to either healthy controls or remission stage in this study. Our observations are in line with the recent results on the importance of IFN-gamma signature and macrophage activation in the pathogenesis of MIS-C and make ferritin and neopterin potentially useful clinical markers to support the diagnosis of MIS-C and to monitor therapy efficacy.

Among children with MIS-C, immunomodulatory therapy with application of intravenous immunoglobulins and corticosteroids show high efficacy^15^ and only a few of the patients required additional interleukine-1 inhibitory treatment^30^. IVIG is a pooled preparation of normal IgG obtained from several thousand healthy donors and is widely used as an immunomodulatory therapy for a number of autoimmune and inflammatory disorders^31^, including Kawasaki disease^32^. The mechanisms of action of IVIG are complex, including several non-exclusive mechanisms affecting soluble molecules and cellular constituents of the immune system. Molecular targets of IVIG therapy depend on the Fc or F(ab')_2_ parts of immunoglobulins, making cell-surface expressed receptors (mainly Fc-receptors) and soluble mediators (including complement) key players. Basta and Dalakas described, that IVIG intercepted the assembly and deposition of sC5b-9 terminal pathway cytolytic complex on capillaries through the formation of complexes between the infused immunoglobulins and C3b, thereby preventing the incorporation of activated C3 molecules into C5 convertase^33^. This observation was confirmed later in experimental stroke models through demonstration of decreased complement mediated neuronal cell death after IVIG treatment^34^. Furthermore, IVIG was reported to have an immediate and long-lasting attenuating effect on complement amplification in vivo^35^, with declining anaphylatoxin C3a and C5a levels after treatment^36^. These results collectively support that the effect of IVIG on the complement system, besides attenuating of pro-inflammatory activation of macrophages^37^, may also contribute to the resolution of inflammatory syndrome in children with MIS-C. Furthermore, with the identification of easily measureable biomarkers of acute versus remission stage of MIS-C our results may fuel further basic research to design experiments investigating the exact chains of events accompanying symptom resolution on a molecular level.

The delayed onset of MIS-C after documented SARS-CoV-2 infection (^14,38^ and Fig. 1), the inability to detect viral RNA in nasal swabs of most patients^38^ with the lack of persisting viral antigens measured even with an ultrasensitive assay^39^ make the contribution of direct viral effects to the development of this condition less probable. Risk factors for PICU treatment in pediatric COVID-19 comprise high degree inflammation, cytopenia, age, comorbidities, and organ dysfunction^40^. Severe MIS-C was indeed linked to similar risk factors like increased concentrations of C-reactive protein, troponin, ferritin, D-dimer, brain natriuretic peptide, or interleukin-6, or reduced platelet or lymphocyte counts in a recent analysis^2^, making contribution of indirect effects, like immune dysregulation^4^ more plausible. The detailed description and characterization of immune dysregulation and autoimmunity in MIS-C identified, among others, autoantibody repertoires against endothelial, mucosal, and immune antigens^4,6^. Whether any of the macrophage or complement activation markers, described earlier or in this study, are suitable to predict the development of MIS-C after COVID-19 exposure, requires further studies.

In summary, the list of complement parameters appropriately supporting the diagnosis and response to therapy in MIS-C includes classical pathway markers C1s-C1-inhibitor and C4a, alternative pathway marker Bb, the global marker C3a, and the terminal pathway marker sC5b-9, together with Factor B, Properdin and C1-inhibitor. For additional clinical laboratory and macrophage activation markers of acute MIS-C we confirmed that C-reactive protein, haptoglobin, troponin, ferritin, D-dimer, pro-BNP, or reduced platelet- or lymphocyte-, and increased neutrophil counts are suitable markers to validate the clinical diagnosis. For the first time, we described that neopterin, a small molecule belonging to the pteridines family, has prominent proficiency as disease activity marker in MIS-C.

## Limitations of the study

Our study has important limitations. This was a prospective, consecutive, non-randomised observational study prone to bias and confounding, and some clinical data were collected retrospectively. However, our study design (by ensuring that all study participants had evidence of prior SARS-CoV-2 infection, by collection of paired samples, by careful sample collection and analytical measurements on the same days for all complement markers and neopterin) helped to reduce the effect of this confounding and bias. The number of MIS-C patients included in this study is fairly low (n = 34), but by the collection of 28 acute (before immunomodulatory therapy) -remission sample pairs with appropriate plasma aliquots for complement analysis, this is among the largest studies reported until now with similar measurements. The effect sizes between healthy controls, acute and remission groups were large enough to yield sufficient power and strongly significant differences even with such low case numbers and with correction for false positive discovery. The healthy control group was used to provide estimates of normal values in the case of variables where reference ranges are not known or not well established. It has to be noted, however, that we did not have a sufficiently large diseased patient control group, therefore we were not able to determine whether the described changes are specific to MIS-C. This should be investigated by further studies. Despite all of our efforts, due to study design and size, observations in this report are to be considered preliminary until independent confirmation.

## Methods

### Materials availability

The applied autoantibodies, commercial assays and further reagents are summarized in Table 2.

**Table 2:** Key resources.

Reagent or Resource	Source	Identifier
**Antibodies**		
Mouse anti-human IgG1Fc-HRP (HP6001)	Southern biotech	Cat# 9054–05
Mouse anti-human IgG2 Fc-HRP (31-7-4)	Southern biotech	Cat# 9060–05
Mouse anti-human IgG3 Hinge-HRP (HP6050)	Southern biotech	Cat# 9210–05
Mouse anti-human IgG4 Fc-HRP (HP6025)	Southern biotech	Cat# 9200–05
Goat anti-human IgG HRP	Southern biotech	Cat# 2040–05
Native human IgG1 protein	abcam	Cat# ab90283
Native human IgG2 protein	abcam	Cat# ab90284
Native human IgG3 protein	abcam	Cat# ab118426
Native human IgG4 protein (not available)	abcam	Cat# ab90286
Anti-sheep red blood cell stroma antibody (rabbit)	Sigma-Aldrich	Cat# S1389
Polyclonal antiserum to human factor B	Quidel	Cat# A311
Polyclonal antiserum to human factor I	Quidel	Cat# A313
Polyclonal antiserum to human C1 inhibitor (C1 esterase)	Quidel	Cat# A300
Polyclonal antiserum to human C1q protein	Quidel	Cat# A301
Monoclonal antibody to human factor H #1	Quidel	Cat# A229
Polyclonal rabbit anti-human C1q complement	Agilent technologies	Cat# A013602
Sheep anti-human factor H	Binding site	Cat# PC030
**Biological samples**		
Bovine serum albumin	Sigma-Aldrich	Cat# A3059
Chemicals, peptides, and recombinant proteins		
Recombinant SARS-CoV-2 spike His protein, CF	R&D systems, biotecne	Cat# 10,549-CV
Recombinant SARS-CoV-2 spike S1 subunit His-tag protein, CF	R&D systems, biotecne	Cat# 10,569-CV-100
Recombinant SARS-CoV-2 spike S2 subunit His-tag protein, CF	R&D systems, biotecne	Cat# 10,594-CV-100
Recombinant SARS-CoV-2 nucleocapsid His protein, CF	R&D systems, biotecne	Cat# 10,474-CV-050
**Critical commercial assays**		
MicroVue Bb Plus EIA Kit	Quidel	Cat# A027
MicroVue sC5b-9 Plus EIA Kit	Quidel	Cat# A020
MicroVue C4d Fragment EIA Kit	Quidel	Cat# A009
MicroVue C4a Fragment EIA Kit	Quidel	Cat# A035
MicroVue C3a Plus EIA Kit	Quidel	Cat# A032
MicroVue C1-Inhibitor Plus EIA Kit	Quidel	Cat# A037
Neopterin ELISA	IBL International GmbH	Cat# RE59321
MASP1-C1-inhibitor complex, Human, ELISA Kit	Hycult biotech	Cat# HK3001*
C1s-C1-inhibitor complex, Human, ELISA Kit	Hycult biotech	Cat# HK399*
Complement factor D, Human, ELISA kit	Hycult biotech	Cat# HK343
Properdin, Human, ELISA kit	Hycult biotech	Cat# HK334
WIESLAB® Complement System MBL Pathway	SVAR life science	Cat# COMPLMP320
WIESLAB® Complement System Alternative Pathway	SVAR life science	Cat# COMPLAP330
GA CoV-2 IgG	GA Generic Assays	Cat# 3920
GA CoV-2 IgM	GA Generic Assays	Cat# 3930
Beckman Coulter C3	Beckman Coulter	Cat# OSR6159
Beckman Coulter C4	Beckman Coulter	Cat# OSR6160
**Deposited data**		
None		
**Software and algorithms**		
GraphPad Prism 9	Graphpad softwares Inc	https://www.graphpad.com/scientific-software/prism/
Statistica 13.5	TIBCO softwares Inc	https://www.tibco.com/
Python 3	Python software foundation	https://www.python.org/
Seaborn 0.11.2	Michael Waskom	https://seaborn.pydata.org/
**Other**		

*not yet available commercially.

Two newly developed immunoassays detecting either human C1s-C1-inhibitor complex or human MASP1-C1-inhibitor complex were used in the study. The assays were developed in cooperation with Hycult Biotech in Uden, The Netherlands, and are not yet available commercially.

Further information and requests for resources and reagents should be directed to and will be fulfilled by the corresponding author.

### Human subjects, ethics declarations, sample collection

Age and sex distribution of the included MIS-C patients and healthy individuals are described in the ‘Results’ section.

The study was conducted in accordance with the Declaration of Helsinki and its subsequent revisions, and was approved by the Hungarian Scientific and Research Ethics Committee (ETT-TUKEB; No. IV/2733–5/2021/EKU). Written informed consent was obtained from the parents of patients and controls.

Blood samples were taken via venipuncture or from a central venous catheter, and were immediately transferred to the processing laboratory, where the cells and the supernatant – serum, citrate- and EDTA-anticoagulated plasma—were separated by centrifugation. Serum and plasma aliquots were prepared, immediately frozen and stored at − 70 °C until the measurements were performed.

### Clinical data collection

Clinical and basic laboratory data were extracted from hospital records and electronic databases.

### Determination of clinical chemistry and inflammatory parameters

Measurement of Neopterin levels was performed using a commercially available assay (IBL International GmbH).

Clinical laboratory tests were performed at clinical laboratories of institutions where the patients were treated. Quantitative determinations of serum total protein and albumin concentrations were performed by colorimetric assays on Roche/Hitachi cobas systems. Serum ferritin and CRP concentrations were determined by immunoturbidimetric assays, whereas serum levels of cardiac troponin T, N-terminal pro-BNP and anti-SARS-CoV-2 antibodies (for diagnostic reasons) were determined by electrochemiluminescent assays on the above mentioned laboratory system. D-dimer levels in citrated plasma were measured by a latex enhanced immunoassay on the ACL TOP Family 50 Series.

Haptoglobin concentration was determined from serum aliquots by immunoturbidimetry on a Beckman Coulter AU Chemistry Analyzer.

### Measurement of complement parameters

Commercially available ELISA kits were used to measure the concentrations of complement factors (factor D, properdin), biomarkers of complement activation (C4a fragment, C4d fragment, C3a fragment, Bb fragment, sC5b-9 complex), as well as lectin and alternative pathway activity and functional C1-inhibitor levels (C1-inhibitor activity). Measurements were conducted according to the manufacturers’ instructions and the ELISA kits concerned are listed with the respective source and identifier number in the key resources table below.

Concentrations of C3 and C4 were measured by immunoturbidimetry on a Beckman Coulter AU Chemistry Analyzer using the respective C3 and C4 reagents (Beckman Coulter, Brea, CA, USA).

Total classical pathway activity was measured by a hemolytic titration test based on Mayer’s method^41^, using sheep erythrocytes sensitized by rabbit anti-sheep red blood cell antibodies. The antigenic concentrations of factor I, factor B and C1-inhibitor were measured by radial immunodiffusion using specific polyclonal antibodies^42^. Complement C1q and factor H concentrations were determined by in-house sandwich ELISAs^42,43^. Antibodies used for the above measurements are listed in the key resources table.

For the determination of complement activation markers (C3a, C4a, C4d, Bb, sC5b-9 and C1s-C1-inhibitor and MASP1-C1-inhibitor complexes) EDTA-anticoagulated plasma samples were used. These plasma samples were thawed under controlled conditions (in a 37 °C water bath just until they were thawed) directly before the measurements. Multiple freeze–thaw cycles were avoided. Serum samples were used for determinations of complement pathway activities, of complement factor and regulator concentrations and of anti-SARS-CoV-2 antibody levels.

### Development of C1s-C1-inhibitor complex and MASP1-C1-inhibitor complex assays

Novel immunoassay detecting levels of human C1s-C1-inhibitor complex and MASP1-C1-inhibitor in vitro were developed at Hycult Biotech in the Netherlands (Uden). The newly developed assays are not yet available commercially. The assays were performed according to manufacturer’s instructions.

In brief, EDTA plasma samples and standards were incubated in wells coated with antibodies recognizing either activated C1s or MASP1. After incubation and washing, wells were incubated with an HRP-labelled antibody detecting bound C1-inhibitor in complex with the respective serine proteases. The amount of bound C1-inhibitor complexes was quantified by incubation with TMB substrate, before the enzymatic reaction was stopped by addition of oxalic acid after 15 min. Afterwards the absorbance was measured at 450 nm on a microplate reader and the concentration of C1-inhibitor complexes was determined based on a standard curve with known concentrations of either C1s-C1-inhibitor or MASP1-C1-inhibitor complex.

### Measurement of anti-nucleocapsid plus anti-spike SARS-CoV-2 IgM or IgG antibodies

The aggregate amount of IgM and of IgG antibodies against SARS-CoV-2 spike (S) and nucleocapsid (Nc) proteins were detected using commercially available assays (GA Generic Assays GmbH, Germany). The cut-off (Co) and binding index (BI) was calculated according to the manufacturer's instructions: [Cut-off (Co) = 0.250 + OD Negative control, BI = OD sample /Co]. Samples with BI above 0.9 were considered positive.

### Determination of anti-nucleocapsid and of anti-spike SARS-CoV-2 IgG levels by research grade ELISA

In-house ELISAs were performed with nucleocapsid (Nc) or full-length spike (Sfull) or spike domain S1 or S2 recombinant proteins. Briefly, recombinant Nc or S were coated on 96-well polystyrene microtiter plates (Greiner Bio-One GmbH, Austria) at a concentration of 1 µg/ml in 100 µl coating bicarbonate buffer (pH9.8) at 4 °C overnight. After blocking with 1% bovine serum albumin (BSA), the plates were washed thoroughly with phosphate-buffered saline (PBS)-Tween. Patient and control sera were diluted 1:25 and loaded on the plates in duplicates. Plates were further incubated for 1 h at room temperature and detected by a goat anti-human IgG secondary antibody labelled with HRP (SouthernBiotech). Absorbance values of samples and positive and negative controls were measured at 450 nm, with a reference wavelength of 620 nm using an automated plate reader (Tecan Group Ltd, Switzerland). Anti-SARS-CoV-2 Sfull, S1, S2 and Nc IgG levels were performed in normalized values (OD sample / OD positive control). Cut-off values were determined by the mean value plus 2 times the standard deviation (SD) of the negative control.

### Determination of IgG subclasses against the full-length spike protein

Briefly, recombinant Sfull or Nc proteins and calibrators of purified human antibodies of each IgG subclass ranging from 1000 to 0 ng/ml were coated on 96-well polystyrene plates (Greiner Bio-One GmbH) overnight. After blocking, serum samples were added to the plate in dilutions between 1:25 and 1:250 and incubated for 1 h at room temperature. The bound antibodies were then detected by HRP-conjugated mouse anti-human subclass specific antibodies. Cut-off values were determined by the mean OD value of 15.6 ng/ml IgG subclasses. Anti-SARS-CoV-2 Sfull IgG1, IgG3 and anti-SARS-CoV-2 Nc IgG1, IgG3 concentrations were plotted in ng/ml.

### Statistical analysis

Most continuous variables did not show normal distributions, so these data were presented as median and interquartile range, and nonparametric tests were used: Mann–Whitney test for the comparison of two independent groups, Wilcoxon matched-pairs ranked sum test for the analysis of paired data, Kruskal–Wallis test with Dunn’s post-test for the comparison of more than two independent groups, and Spearman’s rank correlation test for analyzing the correlations between continuous variables. All applied tests were two-tailed, with the false discovery rate (alpha) of 0.05. The level of significance was corrected by the Benjamini–Hochberg procedure in the case of multiple comparisons to maintain a false discovery rate of 5%. Receiver operating characteristic (ROC) curves were generated and analyzed to determine the area under curve (AUC) and the optimal cut-off points for certain categorical variables. Principal component analysis (PCA) was performed using the PCA package in Statistica.

Statistica 13.5 and GraphPad Prism 9 software were used for statistical analysis. Figure 1B was created using Python 3 programming language and the Seaborn 0.11.2 library.

## Supplementary Information

Supplementary Information.

## Data Availability

All data reported in this paper or any additional information required to reanalyze the data reported in this paper are available from the corresponding author on reasonable request. This paper does not report original code.
