# Supplementary material for: Evidence, detailed characterization and clinical context of complement activation in acute multisystem inflammatory syndrome in children
Source: Sci Rep. 2022 Nov 17;12:19759. doi: 10.1038/s41598-022-23806-5 (PMC9670087; doi:10.1038/s41598-022-23806-5)

## *Supplementary Information*

# Evidence, detailed characterization and clinical context of complement activation in acute multisystem inflammatory syndrome in children

*György Sinkovits*, János Schnur, Lisa Hurler, Petra Kiszel, Zita Z. Prohászka, Pál Sík, Erika Kajdácsi, László Cervenak, Veronika Maráczi, Máté Dávid, Borbála Zsigmond, Éva Rimanóczy, Csaba Bereczki, Loek Willems, Erik J. M. Toonen, Zoltán Prohászka*

***Correspondence:** György Sinkovits, sinkovits.gyorgy@med.semmelweis-univ.hu

**Supplementary Figure S1.** Temporal relationship of SARS-CoV-2 exposure, development of COVID-19 symptoms, development of MIS-C and drawing the first acute sample. Data of 24 patients with acute samples and known exposition are shown, with each line representing a single patient. Ten patients had symptomatic COVID-19 disease, the probable time of SARS-CoV-2 exposure (close contact with infected individuals) could be determined for further 14 patients.

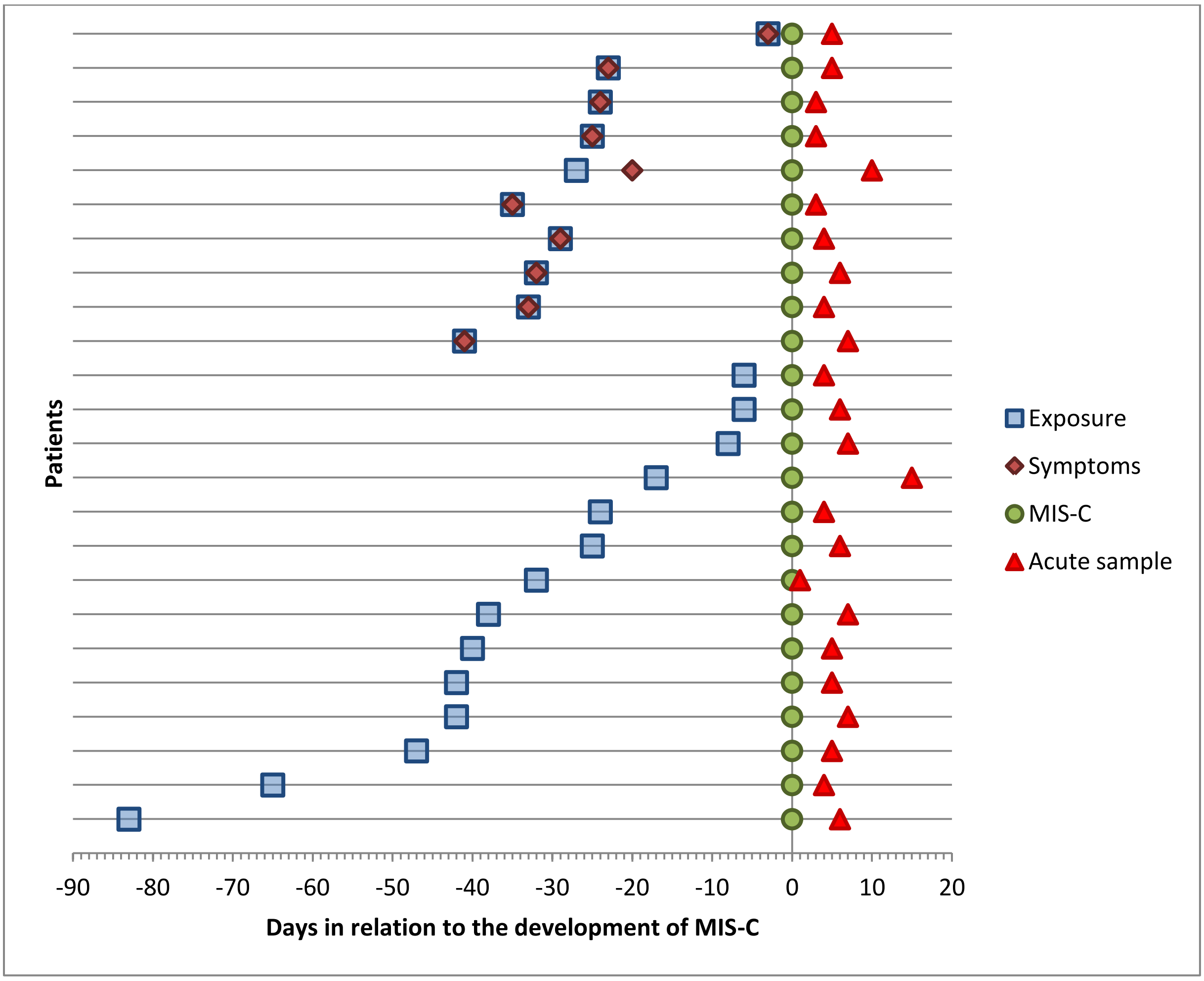

**Supplementary Figure S2.** Clinical and laboratory parameters indicating the severity of MIS-C in groups of patients with and without previous symptomatic COVID-19 disease. (ICU: intensive care unit)

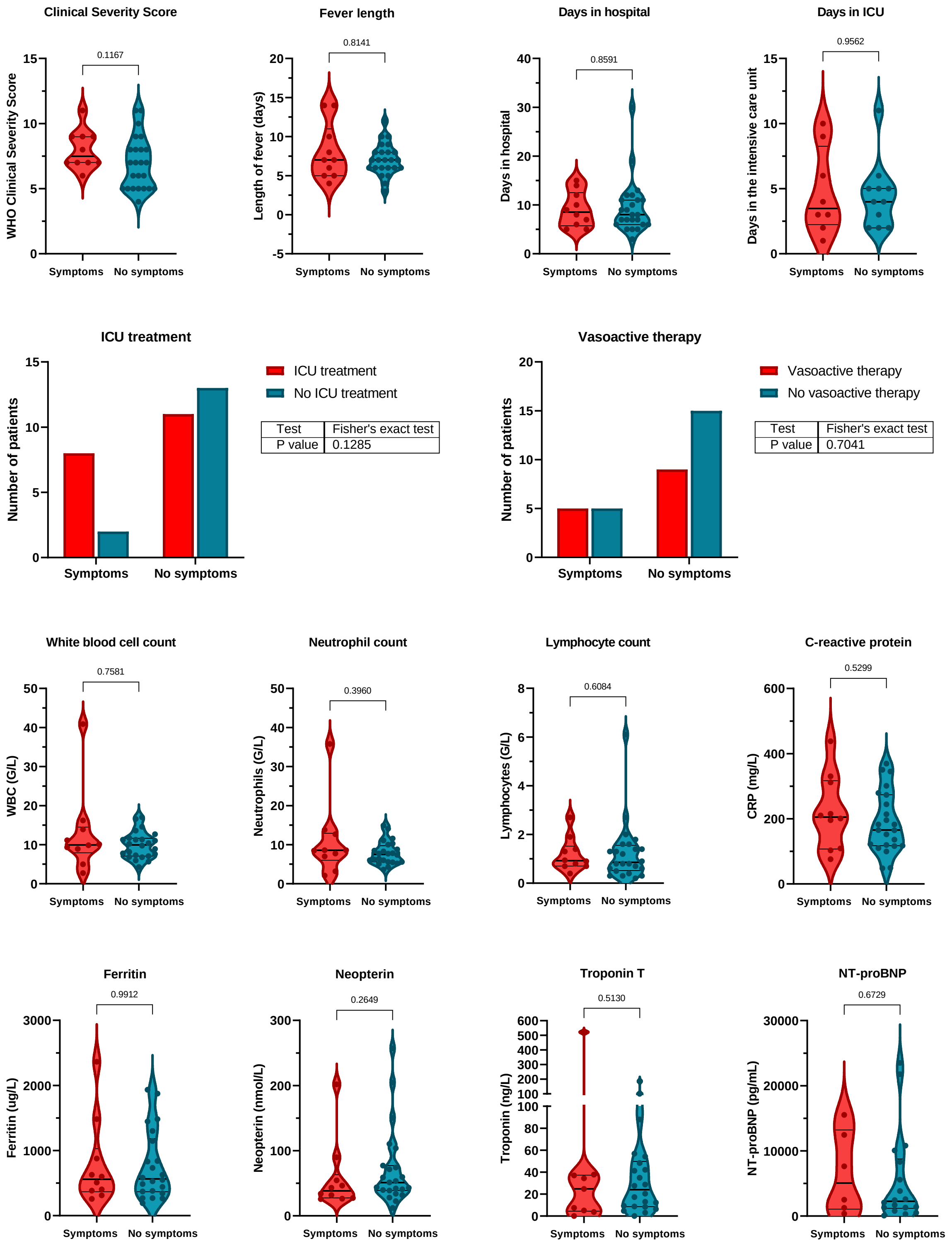

**Figure legend for Supplementary Figures S3-S4:**

**Supplementary Figure S3.** Routine laboratory parameters indicating the severity of MIS-C are shown in acute and remission samples of patients treated or not treated with glycocorticosteroids in addition to intravenous immunoglobulin therapy. Values measured in acute and remission samples of patients receiving steroids are compared to those of acute and remission samples, respectively, of patients who did not receive steroid treatment. Results (p-values) of the Dunn's post hoc tests (following the Kruskal-Wallis test) are indicated in the figure. The limit of significance is p=0.0033 following the Bonferroni-correction for multiple comparisons.

**Supplementary Figure S4.** Relative changes of routine laboratory parameters indicating the severity of MIS-C are shown in acute and remission samples of patients treated or not treated with glycocorticosteroids in addition to intravenous immunoglobulin therapy. To quantify the direction and the extent of changes, values measured in remission samples were expressed as a percentage of those measured in acute samples of the same patients. Results of patients receiving and not receiving steroid therapy were compared by the Mann-Whitney test. The limit of significance is p=0.0033 following the Bonferroni-correction for multiple comparisons.

# Supplementary Figure S3

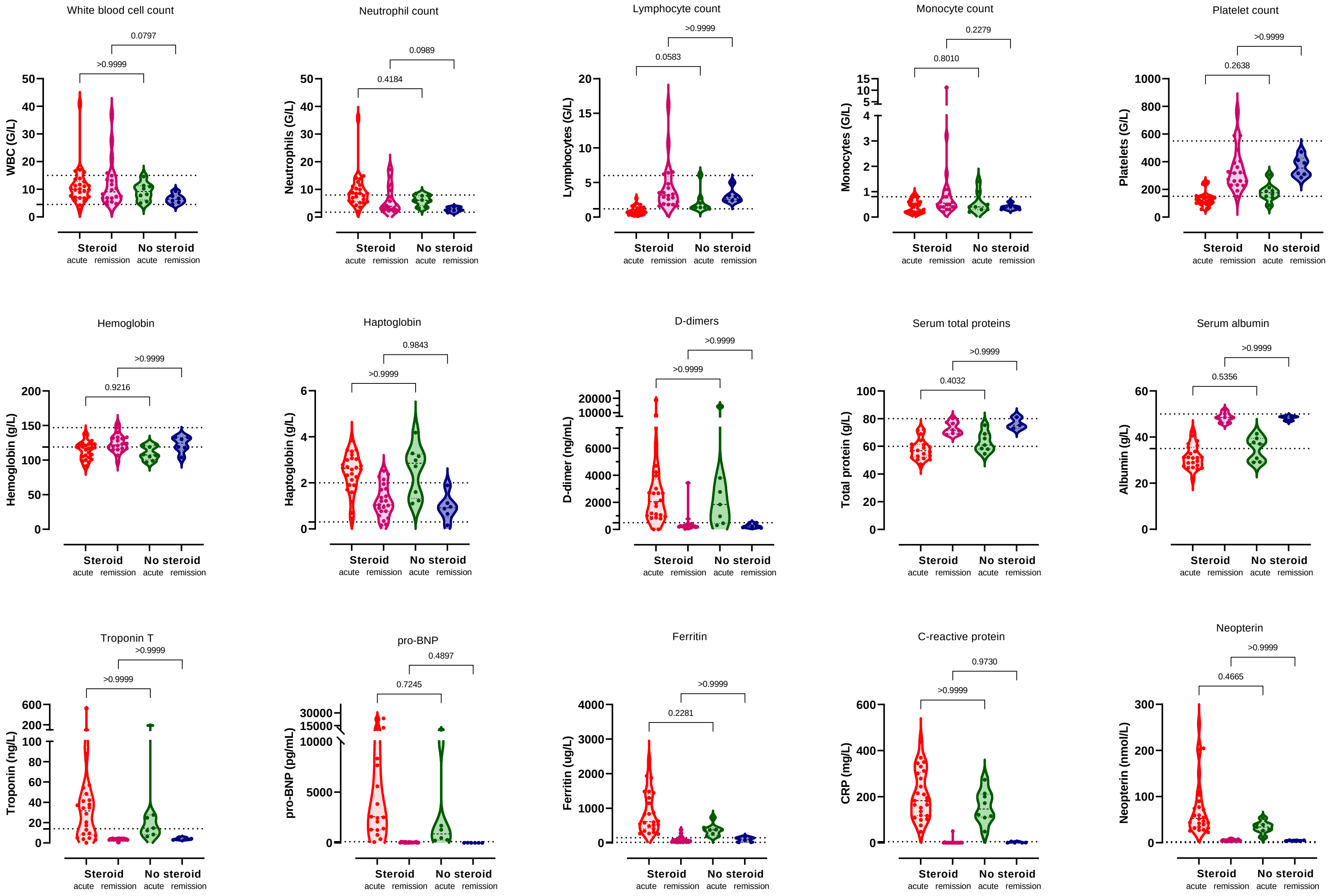

## Supplementary Figure S4

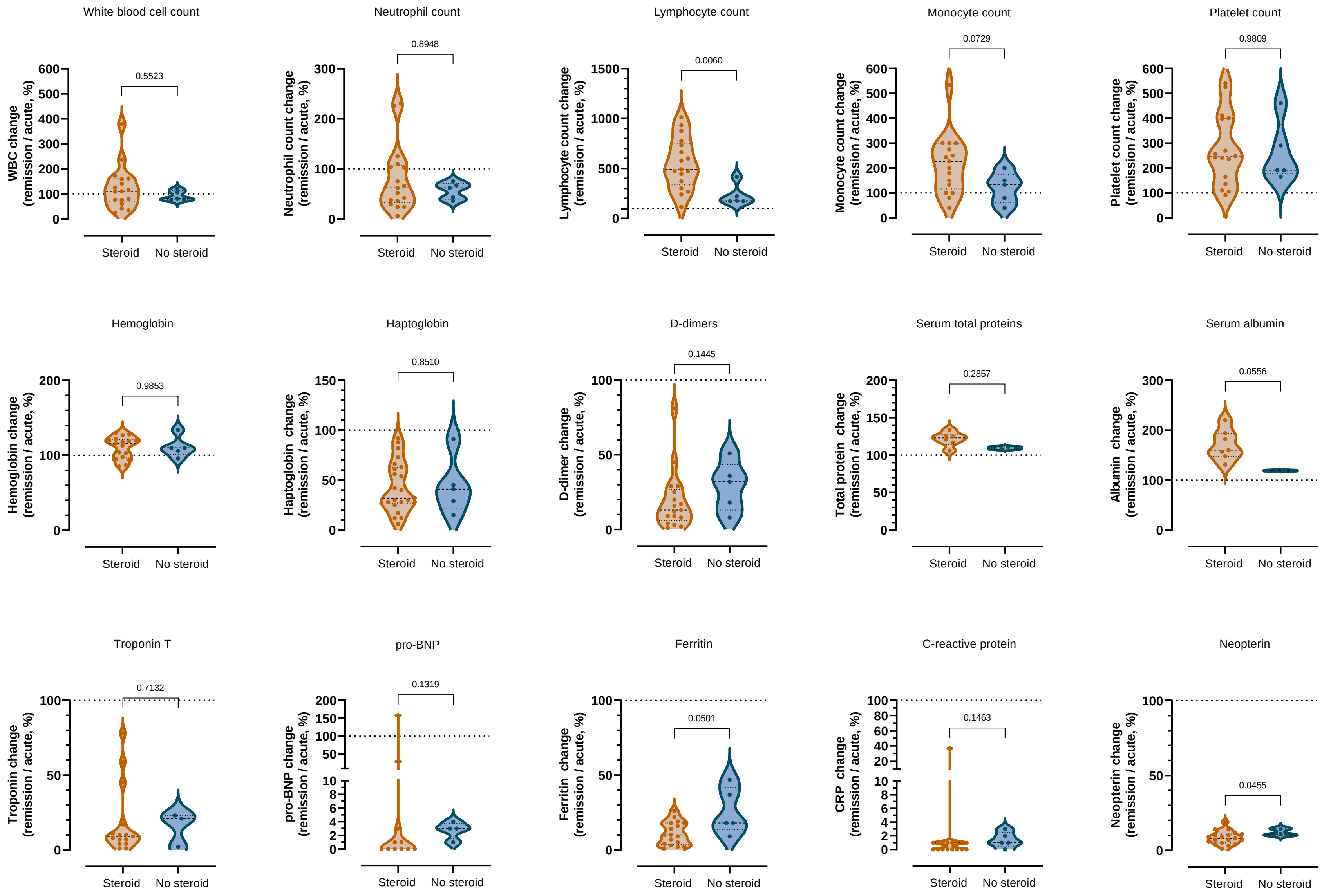

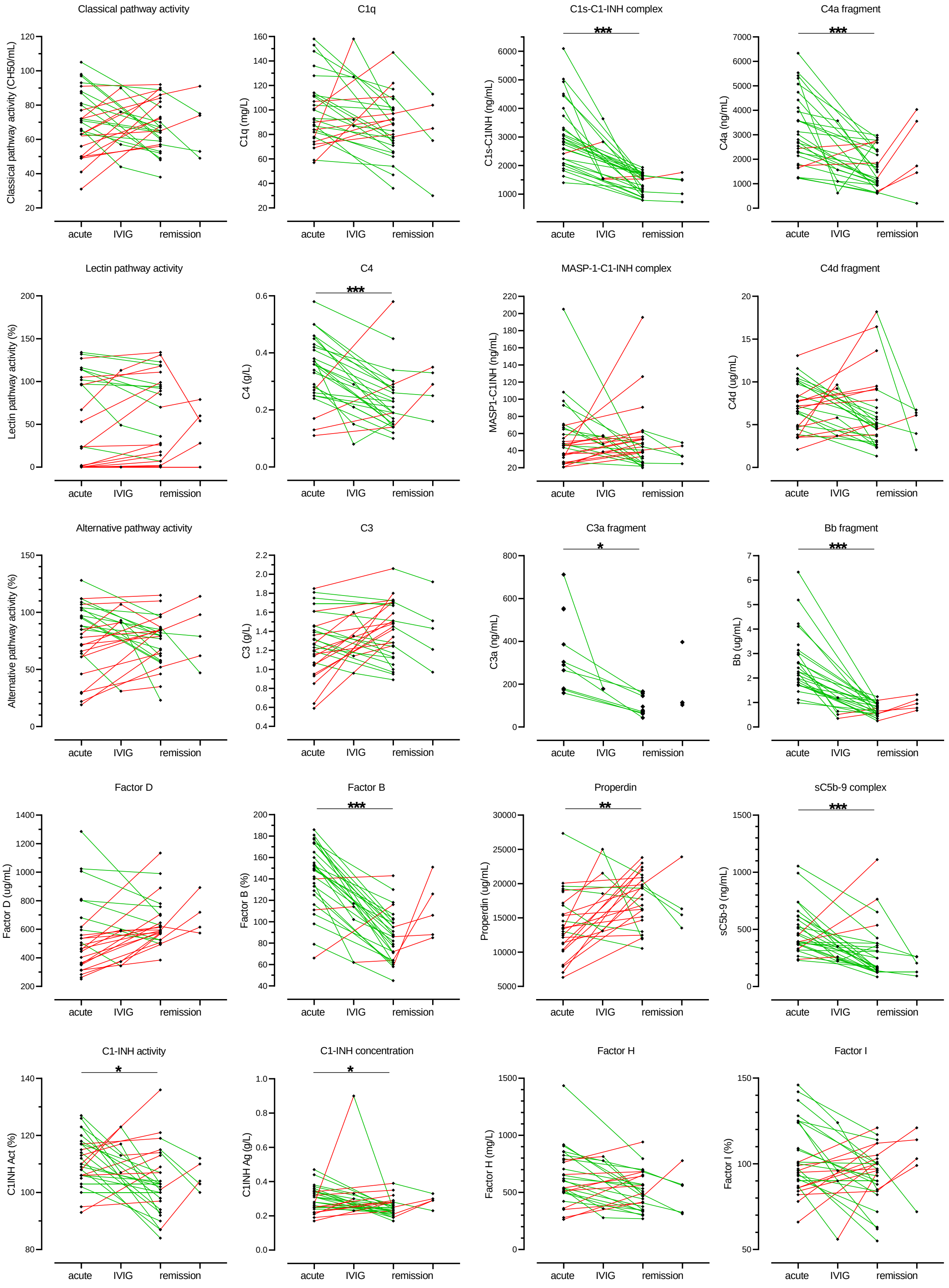


**Supplementary Figure S5.** Complement parameters in paired acute, post-IVIG and remission samples. Results of a single patient are connected with lines: a red line indicates elevation whereas a green line indicates decrease of the given parameter. Acute and remission results were compared by the Wilcoxon rank sum test; symbols (* $p < 0.05$, ** $p < 0.01$, *** $p < 0.001$) indicate significant results after 5% false discovery rate correction using the Benjamini-Hochberg procedure.

**Figure legend for Supplementary Figures S6-S7:**

**Supplementary Figure S6.** Complement parameters – levels of complement components, regulators, activation products and activities of distinct complement pathways – are shown in acute and remission samples of patients treated or not treated with glycocorticosteroids in addition to intravenous immunoglobulin therapy. Values measured in acute and remission samples of patients receiving steroids are compared to those of acute and remission samples, respectively, of patients who did not receive steroid treatment. Results (p-values) of the Dunn's post hoc tests (following the Kruskal-Wallis test) are indicated in the figure. The limit of significance is p=0.0025 following the Bonferroni-correction for multiple comparisons.

**Supplementary Figure S7.** Relative changes of complement parameters are shown in acute and remission samples of patients treated or not treated with glycocorticosteroids in addition to intravenous immunoglobulin therapy. To quantify the direction and the extent of changes, values measured in remission samples were expressed as a percentage of those measured in acute samples of the same patients. Results of patients receiving and not receiving steroid therapy were compared by the Mann-Whitney test. The limit of significance is p=0.0025 following the Bonferroni-correction for multiple comparisons.

## Supplementary Figure S6

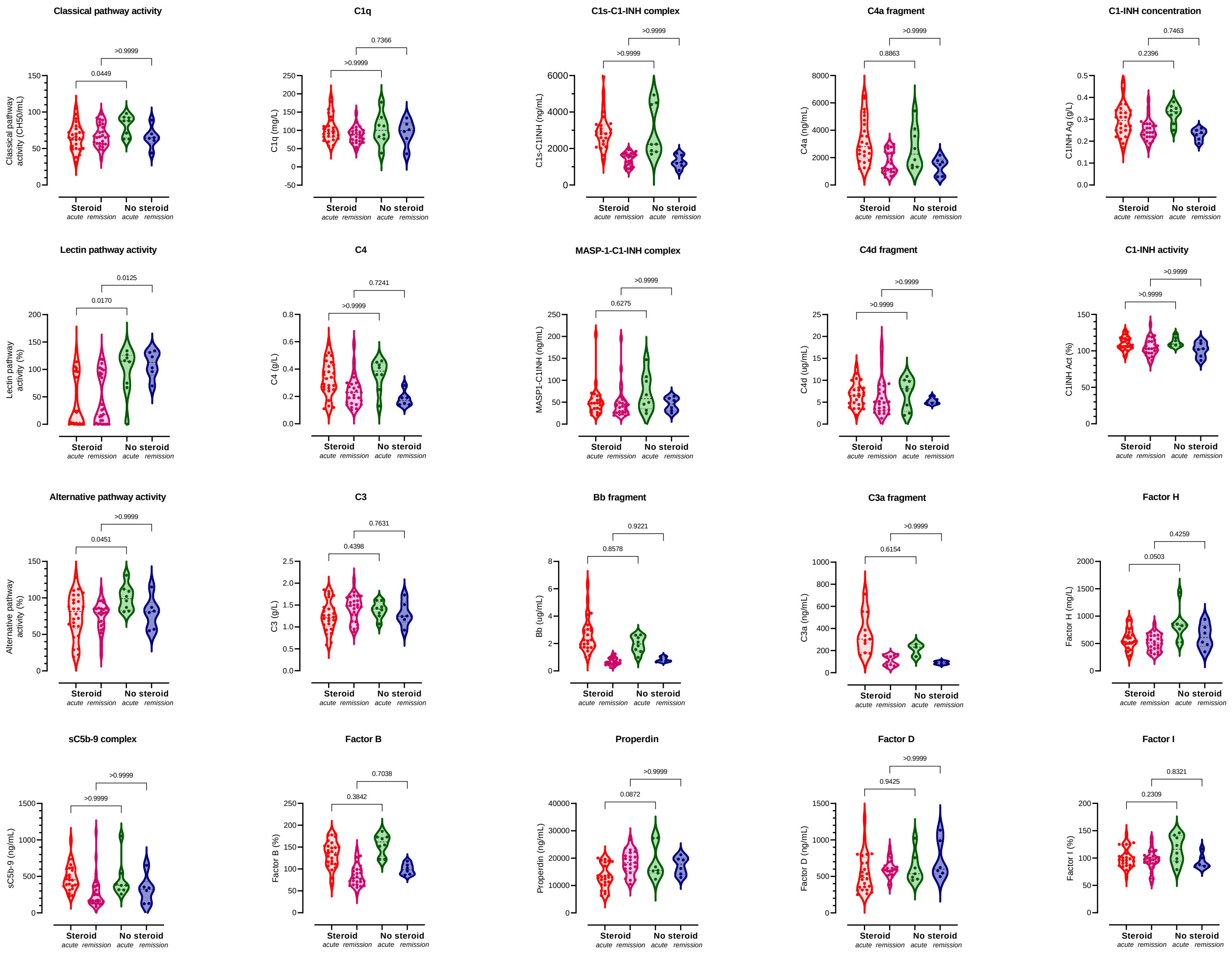

# Supplementary Figure S7

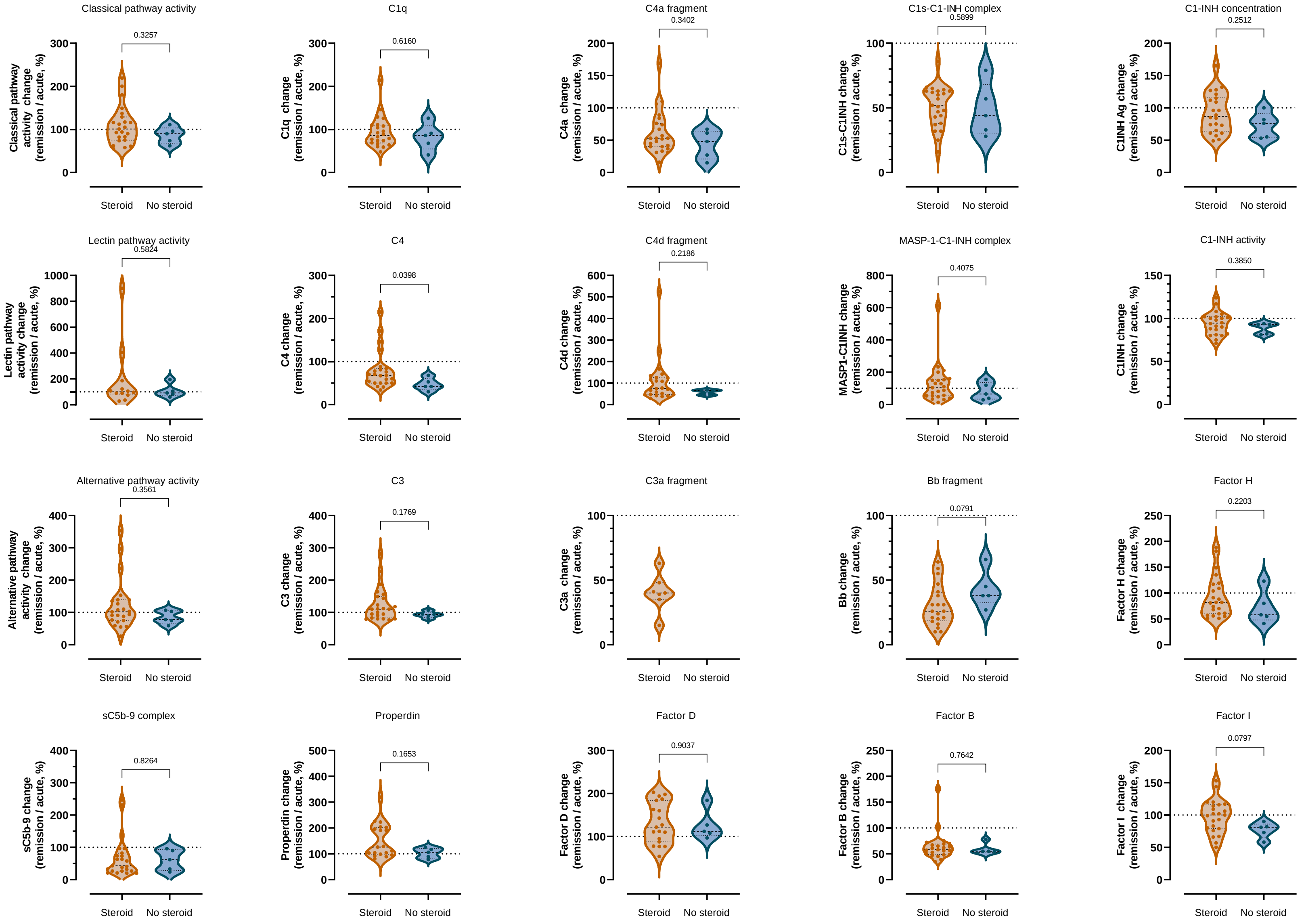

Supplement: Supplementary file 1 — Supplementary Information. [file 41598_2022_23806_MOESM1_ESM.pdf]
